# Mapping of the Sensory Innervation of the Mouse Lung by Specific Vagal and Dorsal Root Ganglion Neuronal Subsets

**DOI:** 10.1523/ENEURO.0026-22.2022

**Published:** 2022-04-13

**Authors:** Seol-Hee Kim, Mayur J. Patil, Stephen H. Hadley, Parmvir K. Bahia, Shane G. Butler, Meghana Madaram, Thomas E. Taylor-Clark

**Affiliations:** Molecular Pharmacology and Physiology, Morsani College of Medicine, University of South Florida, Tampa, FL 33612

**Keywords:** DRG, lung, mapping, nociceptor, sensory nerve, vagal ganglia

## Abstract

The airways are densely innervated by sensory afferent nerves, whose activation regulates respiration and triggers defensive reflexes (e.g., cough, bronchospasm). Airway innervation is heterogeneous, and distinct afferent subsets have distinct functional responses. However, little is known of the innervation patterns of subsets within the lung. A neuroanatomical map is critical for understanding afferent activation under physiological and pathophysiological conditions. Here, we quantified the innervation of the mouse lung by vagal and dorsal root ganglion (DRG) sensory subsets defined by the expression of Pirt (all afferents), 5HT_3_ (vagal nodose afferents), Tac1 (tachykinergic afferents), and transient receptor potential vanilloid 1 channel (TRPV1; defensive/nociceptive afferents) using Cre-mediated reporter expression. We found that vagal afferents innervate almost all conducting airways and project into the alveolar region, whereas DRG afferents only innervate large airways. Of the two vagal ganglia, only nodose afferents project into the alveolar region, but both nodose and jugular afferents innervate conducting airways throughout the lung. Many afferents that project into the alveolar region express TRPV1. Few DRG afferents expressed TRPV1. Approximately 25% of blood vessels were innervated by vagal afferents (many were Tac1+). Approximately 10% of blood vessels had DRG afferents (some were Tac1+), but this was restricted to large vessels. Lastly, innervation of neuroepithelial bodies (NEBs) correlated with the cell number within the bodies. In conclusion, functionally distinct sensory subsets have distinct innervation patterns within the conducting airways, alveoli and blood vessels. Physiologic (e.g., stretch) and pathophysiological (e.g., inflammation, edema) stimuli likely vary throughout these regions. Our data provide a neuroanatomical basis for understanding afferent responses *in vivo*.

## Significance Statement

Activation of airway sensory afferent nerves by physical and chemical stimuli evokes reflex changes in respiratory function. Multiple afferent subsets exist, including those activated by noxious stimuli (so-called “nociceptors”), which have distinct functions. The inappropriate activation of airway afferents, especially nociceptors, in inflammatory/infectious disease contributes to morbidity (e.g., bronchospasm, mucus secretion, cough). Despite extensive electrophysiological characterization of airway afferent subsets, little is known of their innervation patterns. To date, afferent subsets have been qualitatively identified in airway tissue, mostly using immunohistochemistry (IHC; which often lacks specificity and signal strength). Here, we have used Cre-dependent reporter expression to quantify genetically-defined afferent subsets. Thus, we provide a neuroanatomical map of the sensory innervation of conducting airways, alveoli and blood vessels throughout the lung.

## Introduction

The mammalian lung is densely innervated by sensory afferent nerves, whose activation modulates respiratory rhythms and triggers reflex changes in airway function (Coleridge and Coleridge, 1984; Mazzone and Undem, 2016). Most airway sensory nerves are projected from neurons residing in the vagal ganglia, but there is also a small component projected from the dorsal root ganglia (DRGs) neurons. Airway sensory nerves are heterogenous with respect to size, myelination, conduction velocity, stimuli sensitivity, and neuropeptide content.

Critically, studies have identified biochemical and genetic markers of airway sensory subsets that, when activated, evoke specific physiological responses (Ricco et al., 1996; Ho et al., 2001; Undem et al., 2004; Kubin et al., 2006; Nassenstein et al., 2010; Lieu et al., 2011; Chang et al., 2015; Nonomura et al., 2017; Wang et al., 2017; Mazzone et al., 2020; Taylor-Clark, 2021). Lung sensory nerves have been broadly characterized into two groups: (1) myelinated fast conducting Aβ fibers that express the mechanosensitive Piezo2 channel and are activated by physiologically-relevant mechanical forces (inflation and deflation). Activation of these afferents contributes to homeostatic regulation of breathing (so-called “low-threshold mechanoreceptors”); (2) unmyelinated slow conducting C fibers that are activated by noxious stimuli, triggering defensive reflexes such as cough, dyspnea, mucus secretion and bronchospasm (so-called “nociceptors”). The capsaicin-sensitive transient receptor potential vanilloid 1 channel (TRPV1) is a marker of nociceptive neurons and is expressed on almost all airway C fibers (Ricco et al., 1996; Ho et al., 2001; Kollarik et al., 2003; Undem et al., 2004; Hooper et al., 2016). Excessive activation of airway afferents, in particular C fibers, is responsible for many symptoms and outcomes of airway disease caused by inflammation or infectious agents. Indeed, ablation of TRPV1-expressing vagal afferents reduces airway hyperreactivity associated with allergic asthma and increases survival and bacterial clearance in a model of pneumonia (Tränkner et al., 2014; Baral et al., 2018). Thus, airway sensory nerves and their evoked reflexes are therapeutic targets.

The vagal ganglion is actually comprised of two distinct ganglia (nodose and jugular), whose neuronal populations are derived from distinct embryological sources (placodes and neural crest, respectively; Taylor-Clark, 2021). Embryological origin defines gene expression, with selective expression of the transcription factor phox2b, the ion channels P2X_2_ and 5HT_3_, and the neurotrophin receptor TRKB in nodose neurons, and selective expression of the signaling molecule Wnt1, the transcription factor Prdm12 and the neurotrophin receptor TRKA in jugular neurons (Undem et al., 2004; Chuaychoo et al., 2005; Kwong et al., 2008; Nassenstein et al., 2010; Lieu et al., 2011; Nonomura et al., 2017; Kupari et al., 2019; Mazzone et al., 2020; Kim et al., 2020a,b). Furthermore, expression of the tachykinin neuropeptide substance P (Tac1 gene) in TRPV1+ vagal afferents is restricted to jugular neurons. DRG neurons are also derived from the neural crest, and their gene expression resembles jugular neurons (Nassenstein et al., 2010; Usoskin et al., 2015).

Importantly, embryological origin also determines physiological function (Taylor-Clark, 2021), e.g., jugular C fiber activation evokes cough and tachykinin-dependent neurogenic bronchospasm, whereas nodose C fiber activation decreases cough reflex sensitivity and fails to induce neurogenic bronchospasm (Ellis and Undem, 1994; Muroi et al., 2013; Chou et al., 2018). However, heterogeneity in other reflex responses is not so easily explained: in anesthetized guinea pigs, inhalation of capsaicin causes tachypnea, whereas application of capsaicin to the trachea causes bradypnea and intravenous capsaicin causes tachypnea followed by bradypnea (Chou et al., 2008). Furthermore, both the inhalation and intravenous injection of the irritant allyl isothiocyanate (Nassenstein et al., 2008; Nesuashvili et al., 2013) causes parasympathetic-mediated reflex bradycardia in rats, but only the inhalation-evoked reflex is abolished by anesthesia (Hooper et al., 2019). As such, in addition to the jugular versus nodose paradigm that determines nociceptive function, there appears to be further complexity which may depend on anatomically distinct subsets.

Despite extensive characterization of the electrophysiological properties of airway afferent subsets, there is a gap in our understanding of their innervation patterns and how this impacts their sensitivity to physiological and pathophysiological stimuli. Immunohistochemistry (IHC) of lung afferents has yielded conflicting data (Taylor-Clark, 2021) and is hampered by a lack of selective markers and the difficulty in labeling varicosities. Recently, genetically-defined afferents have been identified in the lung using cre-lox reporter systems (Nonomura et al., 2017; Su et al., 2021). Here, we have used the cre-lox system to systematically map the afferent innervation of conducting airways, blood vessels and alveolar regions by genetically-defined vagal and DRG subsets using Pirt^Cre^ (all afferents), TRPV1^Cre^ (nociceptors), Tac1^Cre^ (tachykinergic afferents), and 5HT_3_^Cre^ (nodose afferents).

## Materials and Methods

### Animals and genotyping

All procedures were in accordance with the animal protocol approved by the Institutional Animal Care and Use Committee. Four Cre strains were used: (1) the knock-in TRPV1^Cre^ (B6.129 × 1-Trpv1tm1(cre)Bbm/J, IMSR_JAX:017769, The Jackson Laboratory; Cavanaugh et al., 2011b); (2) the knock-in Tac1^Cre^ (B6.129S-Tac1<tm1.1(cre)Hze/J, IMSR_JAX:021877; The Jackson Laboratory; Harris et al., 2014); (3) the knock-in Pirt^Cre^ (Pirttm3.1(cre)Xzd, kind gift from Xinzhong Dong, Johns Hopkins University; Patel et al., 2011); and (4) the transgenic 5HT_3_^Cre^ (B6.FVB(Cg)-Tg(Htr3a-cre)NO152Gsat/Mmucd, #037089-UCD, Mutant Mouse Resource and Research Centers; Gong et al., 2003). Cre expression of these strains in the nodose and jugular ganglia has previously been characterized (Kim et al., 2020b; Su et al., 2021). In some cases, the Cre strains were crossed with Ai9 mice (B6.Cg-Gt(ROSA)26Sortm9(CAG-tdTomato)Hze/J, IMSR_JAX:007909, The Jackson Laboratory), which express tdTomato in a Cre-sensitive manner from the ROSA26 gene, to produce Tac1-Ai9, Pirt-Ai9 and 5HT_3_-Ai9 mice with cell-specific expression of tdTomato (tdT) via Cre recombination. Both male and female mice (6–12 weeks old) were used for experiments. Offspring were weaned at 21 postnatal days and up to four littermates were housed per cage under normal condition (20°C, a 12/12 h light/dark cycle). Mice were provided with standard rodent chow and water *ad libitum*.

Purification of DNA for genotyping from ear punches was performed using the HotSHOT procedure (Truett et al., 2000). PCR was performed using HotStarTaq DNA Polymerase (QIAGEN). For each TRPV1^Cre^, Tac1^Cre^, and Pirt^Cre^ PCR, 5.8 μl of DNAase/RNase-free distilled H_2_O was mixed with 1.2 μl 10× PCR buffer (QIAGEN), 1 μl of 25 mm MgCl_2_, 1 μl of deoxynucleotide triphosphate mixture (dNTPs; Takara), 0.5 μl for each of the two primers (20 μm) and 2 μl of purified DNA. Separate reactions were used for mutant and wild-type alleles. TRPV1^Cre^ wild-type primers were TTC AGG GAG AAA CTG GAA GAA (forward) and TAG TCC CAG CCA TCC AAA AG (reverse), yielding a 490-bp product. TRPV1^Cre^ mutant primers were GCG GTC TGG CAG TAA AAA CTA TC (forward) and GTG AAA CAG CAT TGC TGT CAC TT (reverse), yielding a 102-bp product. The common reverse primer for Tac1^Cre^ was GCA TAT TTG GCT TTT ACT CTG G; the wild-type forward primer was GCA TGT TTC CTG TTT CGT GA, yielding a 362-bp product; the mutant forward primer was TGG TGG CTG GAC CAA TGT, yielding a 510-bp product. The common reverse primer for Pirt^Cre^ was TCC CTG GGA CTC ATG ATG CT; the wild-type forward primer was CAA CTT TGT GGT ACC CGA AG, yielding a 194-bp product; the mutant forward primer was ATC CGT AAC CTG GAT AGT GAA, yielding a 277-bp product. For each 5HT_3_^Cre^ PCR, 17.5 μl of DNAase/RNase-free distilled H_2_O was mixed with 2.5 μl 10× PCR buffer, 1 μl of 25 mm MgCl_2_, 1 μl of dNTPs, 0.5 μl for each of the two primers (20 μm) and 2 μl of purified DNA. 5HT_3_^Cre^ transgene primers were GTC TGC CTG GGA CAT GAG GTT G (forward) and CGG CAA ACG GAC AGA AGC ATT (reverse), yielding a 208-bp product. Following an initial 3-min denaturation at 95°C, the DNA was amplified for 30 cycles of denaturation at 94°C for 30 s, followed by annealing at 62°C (TRPV1^Cre^ and Tac1^Cre^) or 55°C (Pirt^Cre^ and 5HT_3_^Cre^) for 30 s and then extension at 72°C for 1 min. The final extension period was increased to 5 min; 4 μl of product was mixed with 6 μl of DNAase/RNase-free distilled H_2_O and 2.5 μl 5× DNA Loading Buffer Blue (Bioline) then run on a 1.5% agarose gel with 100-bp Hyperladder. Bands were visualized with a Biorad GelDoc.

### Administration of adeno-associated virus (AAV)

The following AAV were used: (1) AAV9-flex-GFP, with Cre-sensitive enhanced green fluorescent protein (GFP) expression under the control of a cytomegalovirus enhancer fused to the chicken β-actin (CAG) promoter and a woodchuck hepatitis virus posttranscriptional regulatory element (WPRE; 1.9 × 10^13^ GC/ml, #510502, Addgene); (2) AAV9-flex-tdT, with Cre-sensitive tdTomato expression under the control of a CAG promoter and a WPRE (2.1 × 10^13^ GC/ml, #28306, Addgene); (3) rAAV2-flex-tdT, a retrograde tracer with Cre-sensitive tdTomato expression under the control of CAG promoter and a WPRE. The AAV packaging plasmid vector pAAV-CAG-flex-tdTomato-WPRE was purchased from Addgene (#51503) and incorporated into retrograde AAV2 by Boston Children’s Hospital Vector Core (1.5 × 10^13^ GC/ml); (4) AAV9-Flex-Ruby2sm-Flag, with Cre-sensitive Ruby2 spaghetti monster-Flag expression under the control of CAG promoter and a WPRE. The AAV packaging plasmid vector pAAV-CAG-flex-Ruby2sm_Flag-WPRE-SV40 was purchased from Addgene (#98928) and incorporated into AAV9 by Princeton University’s Vector Core (2.0 × 10^13^ GC/ml). Mice were anesthetized with a mixture of ketamine (50 mg/kg) and dexmedetomide (0.5 mg/kg) via intraperitoneal injection. After AAV injection (see below), the mice received atipamezole (5 mg/kg) via subcutaneous injection for rapid recovery. The mice were injected with meloxicam (500 mg/kg, s.c.) as a postanalgesic on the day and 24 h later.

### Unilateral injection of AAV into the vagal ganglia

Using an intraganglionic injection procedure that has been described elsewhere (Kim et al., 2020b), ∼2 cm of incision was made over a shaved superficial portion of the masseter muscle area. The skin was retracted, and the vagus nerve was located. The vagus nerve was separated from the common carotid artery and the anterior laryngeal nerve using a cotton tip. The vagal nodose ganglia was carefully exposed. The virus microinjection assembly consisted of a pulled glass micropipette (∼20-μm tip diameter) attached to a 1-ml syringe via plastic tubing. Micropipettes were filled with AAV9-flex-GFP (1 μl) using capillary force. The tip of the micropipette was gently inserted into the vagal ganglia and then injected by depressing the plunger (∼0.5 Pounds per square inch). For co-injections of AAV9-flex-GFP, AAV9-flex-tdT, and AAV9-Flex-Ruby2sm-Flag, 1 μl of each virus were premixed in a tube, and then a final volume of 1 μl was injected into the ganglia. Three to six weeks later, mice were euthanized by CO_2_ asphyxiation and the ipsilateral and contralateral vagal ganglia and lungs were collected (see below).

### Intraganglionic injection of AAV into the thoracic DRGs

Approximately 4 cm of incision was made over a shaved portion of the mouse back region, just around the neck area. The skin flaps were opened, and the neck muscles were identified. The C7 and T1 vertebra were used as visual guides. We then separated muscle fibers to get to the T1 and T2 intervertebral space. We followed the spinal nerves and identified the beginning of the ganglia. A pulled glass micropipette (∼20-μm tip diameter) was prefilled with 0.75 μl of AAV9-flex-tdT then injected unilaterally into the T1-T3 DRGs. Three to six weeks later, mice were euthanized by CO_2_ asphyxiation and the ipsilateral and contralateral DRG and lungs were collected (see below).

### Intratracheal instillation of rAAV2 for airway-specific nerve tracing

Using a procedure that has been described elsewhere (Kim et al., 2020b), 30 μl of rAAV2-flex-tdT was diluted with 20 μl of minimum essential medium (MEM; Invitrogen) for lung instillation via endotracheal intubation. The mouse was placed on a vertical stand and tongue was gently pulled to find the intubation path. Using an otoscope attached with a speculum, a 20-gauge intravenous catheter (1.5 inches, BD Insyte) was inserted into the trachea. Successful intubation was confirmed by observing respiration-evoked changes in the liquid level in an attached syringe. The syringe was removed and 50 μl of virus/MEM mixture was pipetted into the catheter. The lung was then inflated with a 1-ml syringe filled with 300 μl of air to ensure instillation of the entire volume of the virus/MEM mixture. Three to six weeks later, mice were euthanized by CO_2_ asphyxiation and the vagal ganglia, DRG, brainstem, and lungs were collected (see below).

### Labeling of pulmonary vasculature using liquid latex

Following euthanasia with CO_2_ inhalation, the mouse was perfused with ice-cold PBS through left ventricle. The pulmonary artery was ligated with 5–0 monofilament suture. On the distal side, the artery was cannulated using a 22-gauge blunt needle connected to polyethylene tubing. The inferior vena cava was clamped. Another 22-gauge blunt needle connected to polyethylene tubing was placed in the left ventricle to target the pulmonary vein. A 1-ml syringe was filled with pink and blue colored liquid latex (VWR, 470024-608 and 470024-612) for the pulmonary artery and vein, respectively. The liquid latex for both the artery and vein was simultaneously injected until the liquid latex was visually observed to fill the lung blood vessels. The lung was then dissected out and dropped in ice-cold PBS. After 10 min or washing, the lung was postfixed in ice-cold 3.7% formaldehyde (FA) overnight. The tissue was then washed with ice-cold PBS three times for 30 min. The lung was inflated with 2% low melting agarose via the trachea. After the agarose solution solidified, the lung lobes were separated and 200-μm slices were collected using a vibratome. Lung slices were stained and mounted on a glass slide with VECTASHIELD Antifade Mounting Medium with DAPI (H-1200, Vector Laboratories) for imaging. Brightfield images were obtained using a Keyence microscope (BZ-X700), and fluorescent images were obtained using an Andor Dragonfly spinning disk confocal microscope.

### Tissue collection and IHC

Mice were euthanized by CO_2_ inhalation and transcardially perfused with ice-cold PBS. Vagal ganglia, DRG from T1 to T6 and brainstem were dissected out. Vagal ganglia and DRG were postfixed for 1 h, and the brainstem was postfixed for 4 h in FA at 4°C. These tissues were processed and immunostained as described previously (Kim et al., 2020b). The tissue was washed in PBS to remove residual FA and transferred to 20% sucrose solution for cryoprotection. The tissue was mounted in optimal cutting temperature (OCT) compound and snap frozen in dry ice. Vagal ganglia and DRG were sectioned in 20-μm slices, and brainstem was sectioned in 40-μm slices using a cryostat, and those were collected onto Superfrost plus slides. Slides were air-dried at room temperature in the dark overnight. Slides were washed with PBS three times for 10 min, and tissue was permeabilized with 0.3% Triton X-100 in PBS (PBSTx) for 15 min followed by blocking for 1 h with 1% bovine serum albumin (BSA)/10% donkey serum (DS)/0.3% PBSTx. Tissue was incubated with primary antibodies (Table 1) diluted in blocking buffer overnight at 4°C. After washing with 0.2% Tween 20 in PBS (PBST) three times for 10 min, the tissue was incubated with secondary antibodies (Table 1) in 1% BSA/5% DS in 0.2% PBST for 1 h. The tissue was washed with 0.2% PBST three times for 10 min and then, in some cases, counterstained with NeuroTrace fluorescent Nissl Stain for 1 h at 1:300 dilution in PBS. After washing with PBS, slides were air-dried and mounted with DPX mounting medium (Sigma).

**Table 1:** Antibodies used in this study

Antibodies	Host	Dilution	Catalog number	Source	RRID
Anti-DsRed	Rabbit	1:500	632496	Takara Bio	AB_10013483
Anti-tdTomato	Rat	1:300	EST203	Kerafast	AB_2732803
Anti-GFP	Chicken	1:1000	ab13970	Abcam	AB_300798
Anti-E-cadherin	Rat	1:300	ab11512	Abcam	AB_298118
Anti-α-smooth muscle actin	Goat	1:300	SAB2500963	MilliporeSigma	AB_10603763
Anti-FLAG	Rabbit	1:300	NB600-345	Novusbio	AB_10001331
Anti-FLAG	Goat	1:300	NB600-344	Novusbio	AB_10000565
Anti-CGRP	Rabbit	1:300	C8198	MilliporeSigma	AB_259091
Neurotrace 435/455	N/A	1:300	N21479	Invitrogen	AB_2572212
Neurotrace 500/525	N/A	1:300	N21480	Invitrogen	SCR_013318
Alexa Fluor 488 anti-chicken immunoglobulin	Goat	1:500	A11039	Invitrogen	AB_2534096
Alexa Fluor 488 anti-rabbit immunoglobulin	Donkey	1:500	A21206	Invitrogen	AB_2535792
Alexa Fluor 546 anti-rabbit immunoglobulin	Donkey	1:500	A10040	Invitrogen	AB_2534016
Alexa Fluor 546 anti-rat immunoglobulin	Goat	1:500	A11081	Invitrogen	AB_141738
Alexa Fluor 647 anti-chicken immunoglobulin	Goat	1:500	Ab150171	Abcam	
Alexa Fluor 647 anti-rat immunoglobulin	Goat	1:500	A21247	Invitrogen	AB_141778
Alexa Fluor 647 anti-goat immunoglobulin	Chicken	1:500	A21469	Invitrogen	AB_2535872
Alexa Fluor 647 anti-rabbit immunoglobulin	Donkey	1:500	A31573	Invitrogen	AB_2536183

Lungs were collected and postfixed in 3.7% FA overnight at 4°C with gentle agitation. Lungs were washed with ice-cold PBS three times for 30 min, followed by cryoprotection in 30% sucrose solution until the lung sank to the bottom of the tube. The lungs were flushed with PBS three times and inflated with 2% low melting agarose solution. After the agarose solution had solidified, the lung lobes were separated and snap frozen with OCT compound for cryosection; 80-μm lung slices were collected in cryoprotectant filled well-plates and stored in −20°C. Only ipsilateral lung lobes were included in the analyses in experiments with unilateral intraganglionic AAV injections. Using identical blocking solutions, permeabilizing solutions and antibody solutions (see above), lung slices was washed in PBS three times for 10 min and permeabilized for 20 min. Lung slices were then blocked for 1.5 h followed by primary antibody incubation overnight at 4°C. The slices were then washed three times for 20 min and incubated with secondary antibodies for 2 h. The slices were washed again and mounted onto glass slides with VECTASHIELD Antifade Mounting Medium with DAPI.

### Experimental groups

1. Pirt-Ai9 mice. These mice were divided into two groups. The first group of five mice, which were harvested without latex labeling of the pulmonary vasculature, yielded 10 lung slices. Three lung slices were immunostained for α smooth muscle actin and E-Cadherin, three lung slices were immunostained for E-Cadherin and DAPI and four lung slices were immunostained for CGRP and DAPI. In all 10 slices, native tdTomato was imaged without immunostaining. The second group of three mice, which were harvested following latex labeling of the pulmonary vasculature, yielded six slices. These were immunostained for tdTomato and DAPI. The data from both groups was combined to quantify the overall innervation by tdTomato+ fibers in the Pirt-Ai9 mice.
2. Three Pirt^Cre^ mice were administered rAAV2-flex-tdT via intratracheal instillation, yielding seven lung slices. These were immunostained for tdTomato and DAPI.
3. Three Pirt^Cre^ mice received injection of AAV9-flex-tdT into the DRG and AAV9-flex-GFP into the vagal ganglia, yielding seven lung slices. These were immunostained for E-Cadherin, GFP, tdTomato, and DAPI.
4. Seven 5HT_3_-Ai9 were harvested, yielding 19 lung slices. Six lung slices were immunostained for α smooth muscle actin and E-Cadherin, three lung slices were immunostained for E-Cadherin and DAPI, and 10 lung slices were labeled for DAPI alone. In all 19 slices, native tdTomato was imaged without immunostaining.
5. Tac1-Ai9 mice. These mice were divided into two groups. The first group of six mice, which were harvested without injection of the vagal ganglia, yielded 13 lung slices, which were immunostained for E-Cadherin and DAPI. The second group of five mice, which received injection of AAV9-flex-GFP into the vagal ganglia, yielded 14 lung slices. These were immunostained for GFP and DAPI. For all Tac1-Ai9 lung slices, native tdTomato was imaged without immunostaining. The data from both groups was combined to quantify the overall innervation by tdTomato+ fibers in the Tac1-Ai9 mice.
6. Three Tac1^Cre^ mice received injection of AAV9-flex-tdT into the DRG, yielding five lung slices. These were immunostained for E-Cadherin, tdTomato, and DAPI.
7. TRPV1^Cre^ mice receiving AAV injection into the vagal ganglia were divided into two groups. The first group of seven mice received vagal injection of AAV9-flex-GFP and these yielded 13 lung slices. Eight lungs slices were immunostained for GFP, E-Cadherin, and DAPI, and five lung slices were immunostained for GFP, CGRP, E-Cadherin, and DAPI. The second group of three mice received vagal injection of AAV9-flex-GFP, AAV9-flex-tdT, and AAV9-Flex-Ruby2sm-Flag, and these yielded six lung slices that were immunostained for GFP, tdTomato, Flag, and DAPI. The data from both groups were combined to quantify the overall innervation by GFP+ fibers in the TRPV1^Cre^ mice receiving AAV injection into the vagal ganglia.
8. Four TRPV1^Cre^ mice were administered rAAV2-flex-tdT via intratracheal instillation, yielding nine lung slices. These were immunostained for tdTomato and DAPI.
9. Three TRPV1^Cre^ mice received injection of AAV9-flex-tdT into the DRG, yielding three lung slices. These were immunostained for E-Cadherin, tdTomato, and DAPI.
10. Controls: three lung slices from 5HT_3_-Ai9 mice were used as controls for immunostaining for E-Cadherin and α smooth muscle actin (primary antibody was omitted). In addition, three lung slices from three TRPV1^Cre^ mice that had not received AAV9 injections were immunostained for GFP, tdTomato, and Flag expression.

### Imaging and data analysis

Images were taken with Andor Dragonfly spinning disk confocal microscope equipped with a Zyla 4.2 PLUS sCMOS camera (2048 × 2048 pixels with 6.5-μm pixel size). The pinhole size was 25 μm. We used either a 10× UPLSAPO (0.4 NA), a 20× UPLSAPO (0.75 NA), or a 40× UPLSAPO (1.25 NA, silicone oil immersion) objective was used, depending on the study. Fluorophores were excited by laser wavelengths at 405, 488, 561, or 637 nm. Z-stacked multi-tile images were stitched using either Fusion software or Imaris Stitcher. All 3D images were further processed using Imaris software. Nonconsecutive lung slices were imaged. In most cases the entire slice was imaged. In many cases, staining of E-Cadherin, a marker of airway epithelial cell adherens junctions, was used to identify conducting airways from blood vessels. Nevertheless, conducting airways and vessels could be easily distinguished by DAPI staining: conducting airways are comprised of a compact monolayer of epithelial cells surrounded by lamina propria and smooth muscle compared with diffused endothelial and muscle cells in vessels. All identified conducting airways and vessels had their diameter measured (using the shortest length across the lumen). We then assessed the presence of reporter+ fibers innervating each airway/vessel. This analysis did not distinguish between the density of the fibers or branches. We also determined whether the reporter+ fiber projected away (>20 μm) from the conducting airway or blood vessel into the alveolar region. The maximal straight-line distance projected by the fiber was recorded. Efforts were made to identify nerve terminals within the physical slice, but terminals were not systematically quantified. For some analyses, airways and vessels were grouped into three categories based on diameter: small (0–175 μm), medium (176–375 μm), and large (376 μm and greater).

Control experiments (see study group 10) indicated that reporter expression required AAV-mediated or ROSA26-mediated expression. For experiments involving unilateral intraganglionic AAV injections (vagal, DRG, or both), lung data were only included in the analysis if the injections induced selective and widespread reporter expression in neurons within the respective ipsilateral ganglia. Brightfield images (Keyence) of lung slices from Pirt-Ai9 mice with liquid latex injected into the pulmonary artery (pink) and pulmonary vein (blue) were overlayed with their corresponding confocal fluorescence images (Andor) to determine the Pirt+ fiber innervation of identified arteries and veins. In CGRP-stained lung slices, we identified CGRP+ epithelial cell clusters, termed neuroepithelial bodies (NEBs). The diameter of the conducting airway at the location of the NEB and the number of CGRP+ (and, in some cases, Pirt+) cells within the cluster was recorded. In addition, we analyzed the presence of CGRP+ and reporter+ fibers innervating the NEB. This was defined as a nerve fiber within 10 μm of the NEB. All data were analyzed in GraphPad Prism 9. Groups were compared with the nonparametric Mann–Whitney two-tailed *U* test, *p* < 0.05 was considered significant.

## Results

### Comparison of lung structures innervated by specific sensory nerve subsets

**Figure 1.**
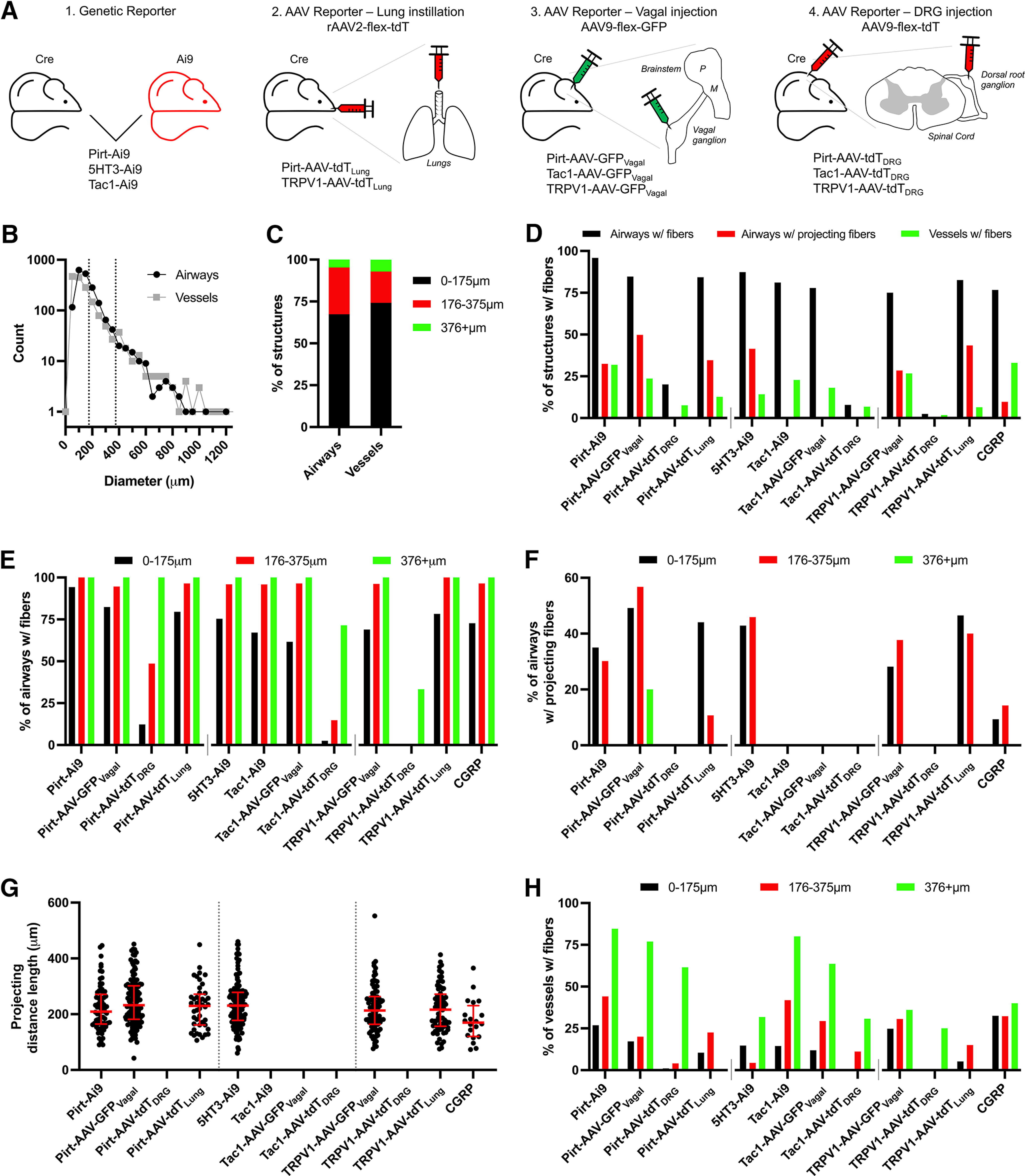
Comprehensive quantification of lung innervation patterns by specific sensory nerve subsets. ***A***, Four approaches used to label specific sensory populations with the fluorescent reporters GFP or tdTomato. ***B***, Histogram of the diameters of conducting airways and blood vessels analyzed. Dotted lines denote 175 and 375 μm. ***C***, Relative quantification of the diameters of conducting airways and blood vessels clustered into “small,” “medium,” and “large” groups. ***D***, Overall quantification of innervation by specific sensory nerve subsets: % of conducting airways with fibers (black), with fibers that project out to the alveolar region (red), and blood vessels with fibers (green). ***E***, % of conducting airways with fibers of specific afferent subsets, clustered by airway diameter. ***F***, Percentage of conducting airways with fibers of specific afferent subsets that project out to the alveolar region, clustered by airway diameter. ***G***, Distance projected from the conducting airway into the alveolar region by fibers of specific afferent subsets (red bars denote median with interquartile range). ***H***, Percentage of blood vessels with fibers of specific afferent subsets, clustered by airway diameter. Data are derived from Pirt-Ai9 [*n* = 8 mice, 219 conducting airways (CA), 270 blood vessels (BV)], Pirt-AAV-GFP_Vagal_ and Pirt-AAV-tdT_DRG_ (*n* = 3 mice, 229 CA, 131 BV), Pirt-AAV-tdT_Lung_ (*n* = 3 mice, 127 CA, 134 BV), 5HT_3_-Ai9 (*n* = 7 mice, 294 CA, 211 BV), Tac1-Ai9 mice (*n* = 11 mice, 281 CA, 259 BV), Tac1-AAV-GFP_Vagal_ (*n* = 5 mice, 208 CA, 188 BV), Tac1-AAV-tdT_DRG_ (*n* = 3 mice, 153 CA, 118 BV), TRPV1-AAV-GFP_Vagal_ (*n* = 10 mice, 313 CA, 288 BV), TRPV1-AAV-tdT_Lung_ (*n* = 4 mice, 161 CA, 139 BV), TRPV1-AAV-tdT_DRG_ (*n* = 3 mice, 82 CA, 57 BV), and CGRP (*n* = 6 mice, 296 CA, 279 BV).

We have used a series of cre recombinase-expressing mouse strains (Pirt^Cre^, 5HT_3_^Cre^, Tac1^Cre^, and TRPV1^Cre^) to identify specific sensory nerve subsets innervating the mouse lung. Cre expression within specific neuronal populations within the sensory ganglia for each of these strains has been previously characterized (Gong et al., 2003; Cavanaugh et al., 2011b; Patel et al., 2011; Harris et al., 2014; Kim et al., 2020b; Su et al., 2021). Cell-specific expression was visualized by Cre-driven fluorescent reporter expression (typically GFP or tdTomato) under the control of either the endogenous ROSA26 gene (Ai9 mouse) or AAV instilled into the lungs or unilaterally injected into sensory ganglia (vagal ganglia or DRG; Fig. 1*A*). Ipsilateral lung slices were sectioned, stained for specific markers and imaged. In some experiments the expression of the sensory neuropeptide CGRP was also determined using IHC. Conducting airways (bronchi and bronchioles) and blood vessels >25 μm in diameter were analyzed (Fig. 1*B*) and stratified into three groups: 0–175 μm (small), 176–375 μm (medium), and >376 μm (large; Fig. 1*C*). Each structure was analyzed for the presence of (1) reporter+ fibers within close proximity (<40 μm) to the epithelium/endothelium, and (2) reporter+ fibers that project away from the structure into the alveolar regions. Generally, >75% of conducting airways had reporter+ fibers (Fig. 1*D*), although this proportion decreased for the smaller conducting airways (Fig. 1*E*). Only a small subset of conducting airways had reporter+ fibers that projected into the alveolar region (Fig. 1*D*), and this only occurred for conducting airways <375 μm in diameter (Fig. 1*F*). There was little difference between the distance that reporter+ fibers from the different strains projected into the alveolar region (Fig. 1*G*). A small subset of blood vessels had reporter+ fibers (Fig. 1*D*), and this proportion tended to decrease for the smaller blood vessels (Fig. 1*H*). Of the 1619 blood vessels analyzed, none had reporter+ fibers that projected into the alveolar regions, and so this analysis was not presented. For some unilateral intraganglionic AAV injections, contralateral lung slices were studied. While some reporter+ fibers were observed, these were very limited in number and not systematically analyzed further.

### Pirt^Cre^

**Figure 2.**
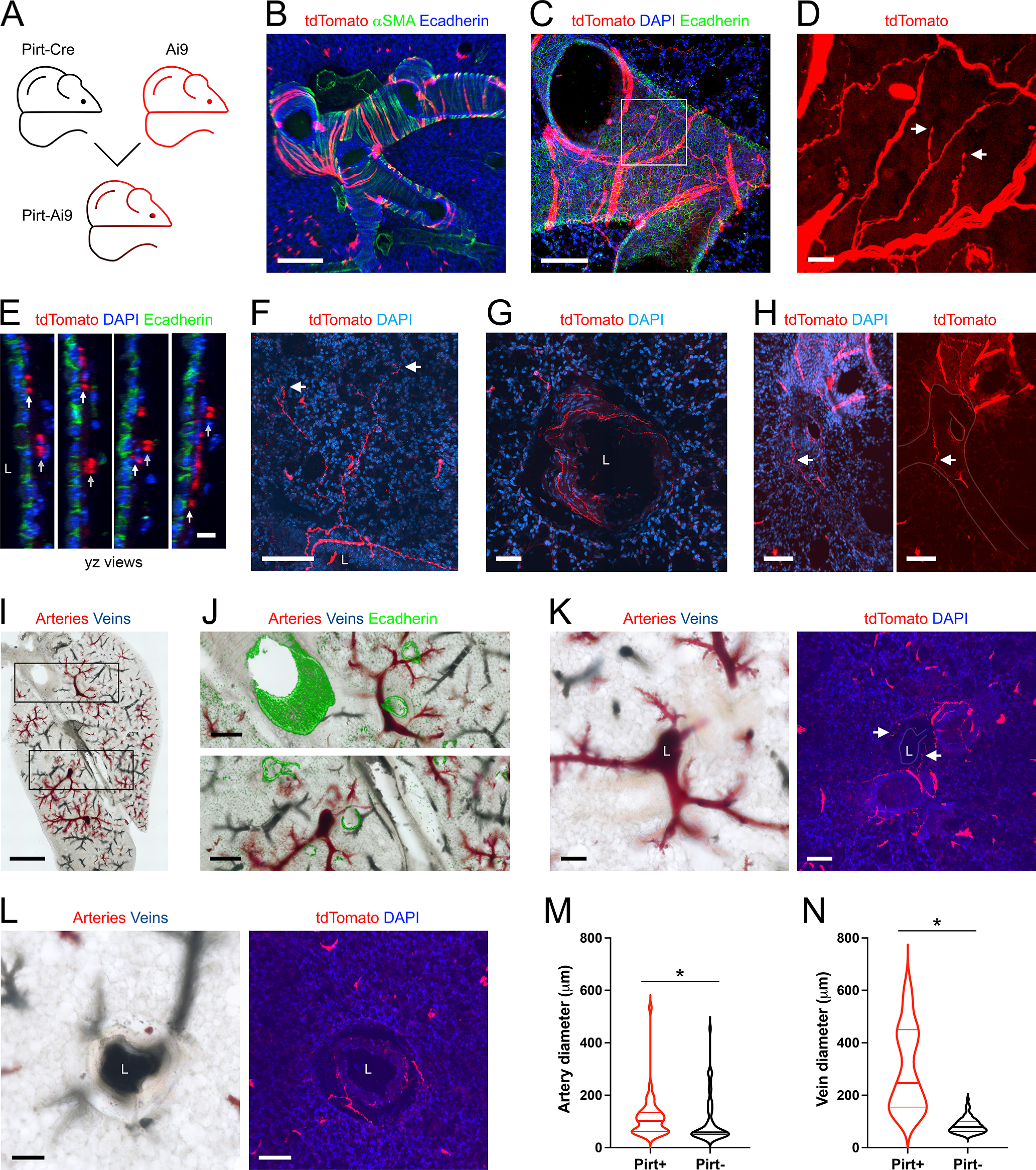
Mapping the lung innervation by Pirt+ nerves. ***A***, Approach for labeling all Pirt+ afferents with tdTomato. ***B***, Lung slice stained for α smooth muscle actin (αSMA, green) and E-Cadherin (blue) showing tdTomato expression (red) in some airway smooth muscle cells. ***C***, Lung slice stained for E-Cadherin (green) and DAPI (blue) showing tdTomato-expressing nerves (red) innervating a large conducting airway. ***D***, Higher magnification of white box in ***C*** (red channel only), with identified tdTomato+ nerve terminals (white arrows). ***E***, *yz* projections of ***C***, showing separate layers of tdTomato+ innervation: individual fibers in close proximity to the epithelial layer (white arrows); thicker cables (comprised of multiple fibers) within the outer smooth muscle layer (gray arrows). ***F***, Lung slice stained for DAPI (blue) showing a tdTomato+ fiber (red) projecting from a conducting airway into the alveolar region, with identified nerve terminals (white arrows). ***G***, Lung slice stained for DAPI (blue) showing a tdTomato+ fiber (red) innervating a blood vessel. ***H***, Lung slice stained for DAPI (blue, left panel only) showing a tdTomato+ (red) fiber (white arrow) innervating a blood vessel (outline superimposed by gray line). ***I***, Brightfield image of lung slice from Pirt-Ai9 with latex labeling of pulmonary arteries (pink latex) and pulmonary veins (blue latex). ***J***, Higher magnification of black boxes in ***I***, with overlay of E-Cadherin staining (green). ***K***, Brightfield image (left) and fluorescent image (right) stained for DAPI (blue), with tdTomato+ (red) fibers (white arrows). ***L***, Brightfield image (left) and fluorescent image (right) stained for DAPI (blue) with tdTomato+ (red) fibers. ***M***, ***N***, Violin plots for the diameters of arteries (***M***) and veins (***N***) with (red) or without (black) tdTomato+ fiber innervation. Data are derived from 3 Pirt-Ai9 mice (101 pulmonary arteries, 104 pulmonary veins). * denotes significant difference between groups (Mann–Whitney two-tailed *U* test, *p* < 0.05, see text for precise values). In some images, lumens are denoted by “L.” Scale bars denote 1 mm (***I***), 300 μm (***J***), 200 μm (***B***), 100 μm (***C***, ***F***, ***H***, ***K***, ***L***), 50 μm (***G***), 15 μm (***D***), or 10 μm (***E***).

**Figure 3.**
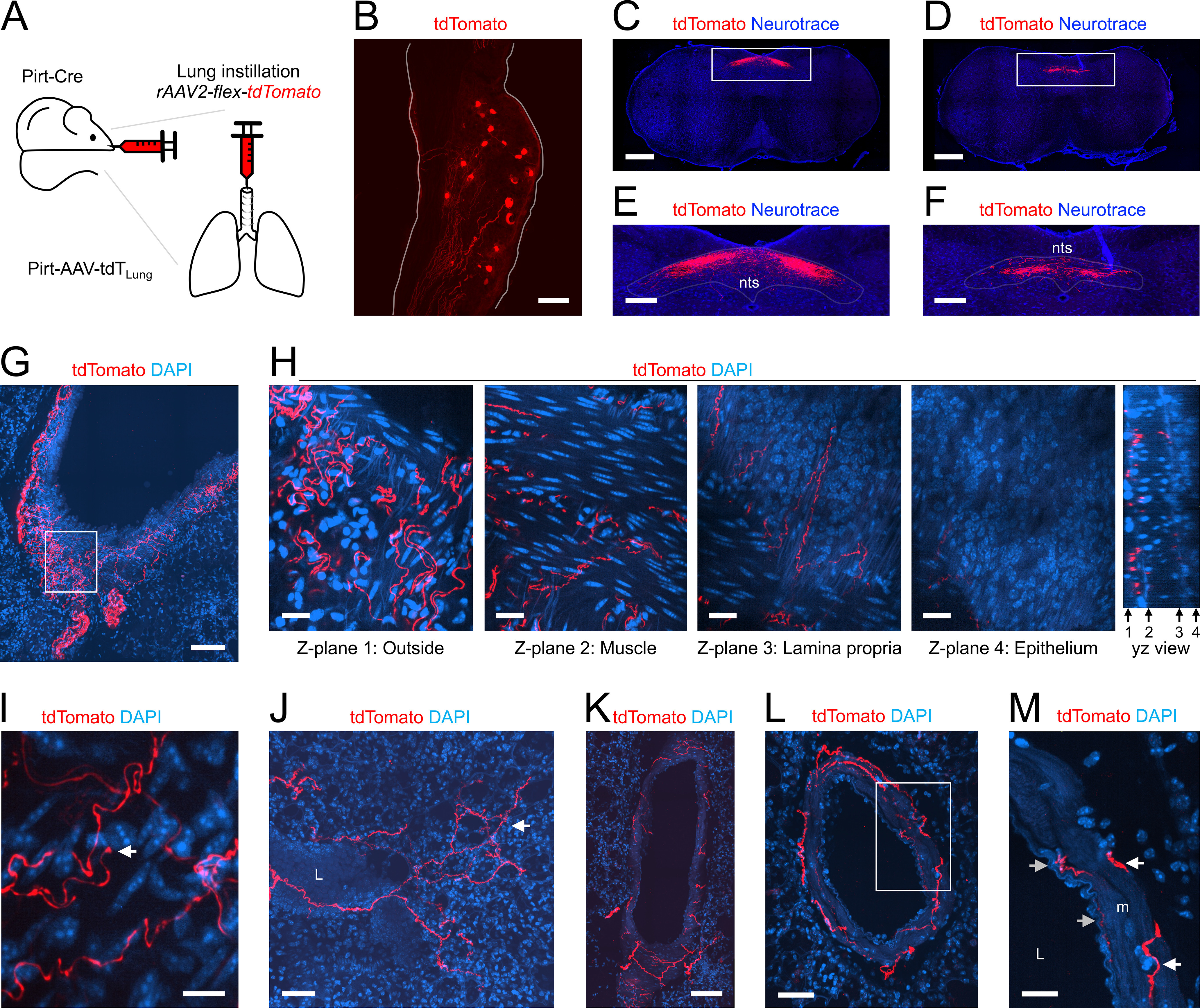
Mapping the lung innervation by Pirt+ nerves labeled by lung instillation of rAAV2-flex-tdTomato. ***A***, Approach for labeling Pirt+ afferents innervating the lungs. ***B***, Vagal ganglia showing lung-labeled tdTomato+ (red) neurons. ***C***, ***D***, Coronal slices of brainstem stained for Neurotrace (blue) showing central projections of lung-labeled tdTomato+ (red) afferents. Slices were located 600 μm (***C***) and 800 μm (***D***) caudal to obex. ***E***, ***F***, Higher magnification of white boxes in ***C***, ***D***, respectively, highlighting the nTS. ***G***, Lung slice stained for DAPI (blue) showing tdTomato-expressing nerves (red) innervating a large conducting airway. ***H***, Individual *z*-planes (1–4, left) and the corresponding *yz* view (right) of the white box in ***G***, at higher magnification. Conducting airway layers were determined by DAPI staining. ***I***, Lung slice stained for DAPI (blue) showing an identified tdTomato-expressing (red) nerve terminal (white arrow) innervating a conducting airway. ***J***, Lung slice stained for DAPI (blue) showing a tdTomato+ fiber (red) projecting from a conducting airway into the alveolar region, with identified nerve terminals (white arrow). ***K***, ***L***, Lung slice stained for DAPI (blue) showing a tdTomato+ fiber (red) innervating a blood vessel. ***M***, Higher magnification of white box in ***L***, with tdTomato+ fibers (red) innervating the inner (gray arrows) and outer layers (white arrows) of the muscle (m). In some images, lumens are denoted by “L.” Scale bars denote 500 μm (***C***, ***D***), 200 μm (***E***, ***F***), 100 μm (***B***, ***G***, ***K***), 50 μm (***J***, ***L***), 20 μm (***H***, ***M***), or 10 μm (***I***).

**Figure 4.**
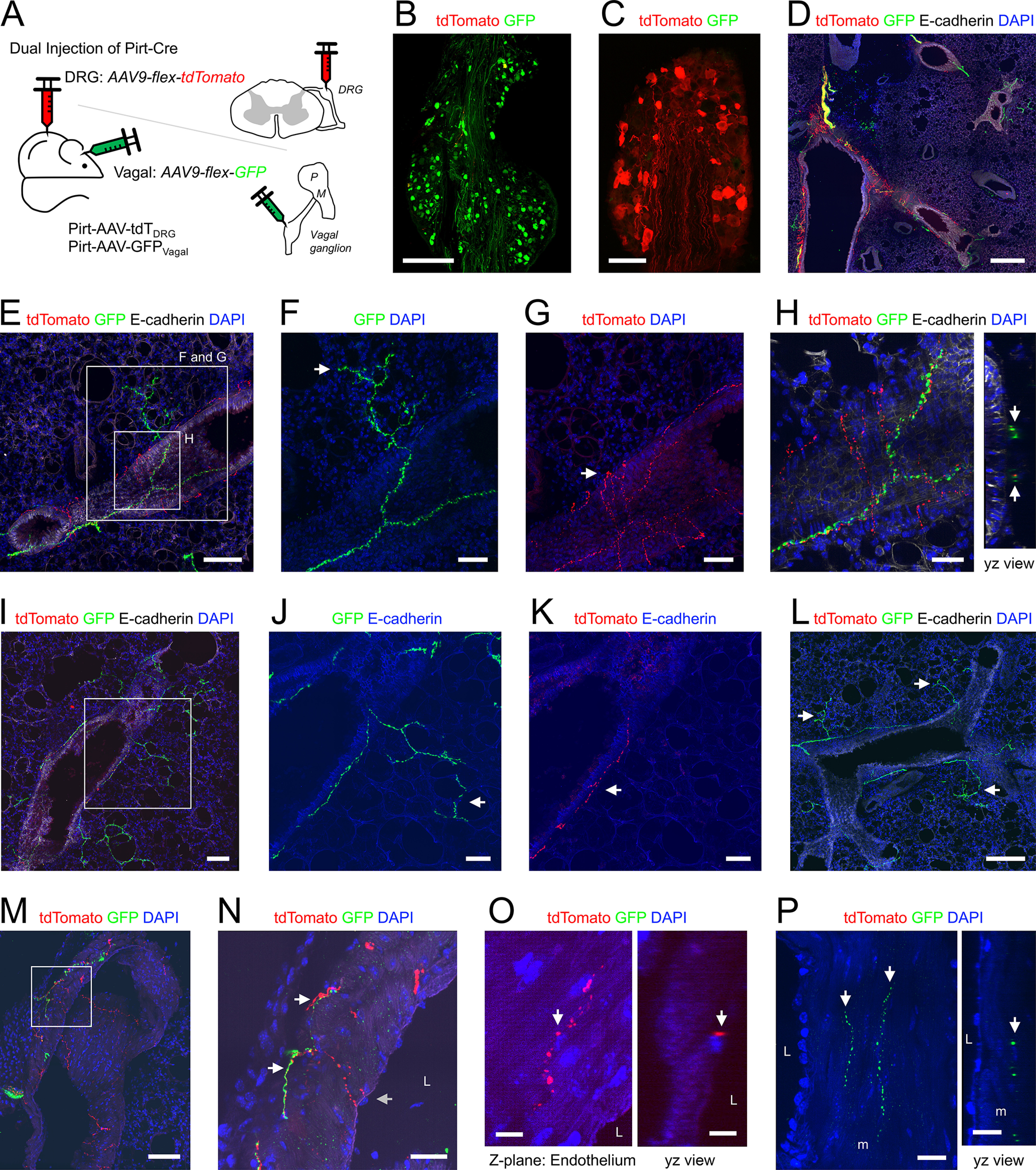
Mapping the lung innervation by Pirt+ fibers projected from DRG and vagal ganglia. ***A***, Approach for labeling Pirt+ neurons in the vagal ganglia with GFP in the DRG with tdTomato. ***B***, Vagal ganglia showing GFP+ (green) neurons. ***C***, DRG showing tdTomato+ (red) neurons. ***D***, ***E***, ***I***, Lung slice stained for E-cadherin (white) and DAPI (blue) showing tdTomato+ (red) and GFP+ (green) fibers. ***F***, Higher magnification of white box in E showing GFP+ fiber projecting from a conducting airway into the alveolar region (white arrow). ***G***, Higher magnification of white box in ***E*** showing tdTomato+ fiber confined to the conducting airway (white arrow). ***H***, Higher magnification of white box in ***E*** showing GFP+ and tdTomato+ fibers beneath the epithelium of a conducting airway are in close proximity. ***J***, Higher magnification of white box in ***I*** showing GFP+ fiber projecting from a conducting airway into the alveolar region (white arrow). ***K***, Higher magnification of white box in ***I*** showing tdTomato+ fiber confined to the conducting airway (white arrow). ***L***, Lung slice stained for E-cadherin (white) and DAPI (blue) showing no tdTomato+ (red) fibers, but many GFP+ (green) fibers innervating the conducting airways and projecting into the alveolar region (white arrows). ***M***, Lung slice stained for DAPI (blue) showing tdTomato+ (red) and GFP+ (green) fibers innervating a blood vessel. ***N***, Higher magnification of white box in ***M***, showing tdTomato+ fibers (gray arrow) extending to the inner muscle layers of the vessel whereas GFP+ fibers innervate the outer layers only (white arrows). ***O***, Lung slice stained for DAPI (blue) lacking GFP+ (green) fibers but showing a tdTomato+ (red) fiber in close proximity to a DAPI+ cell (presumed endothelial cell) adjacent to the blood vessel lumen. ***P***, Lung slice stained for DAPI (blue) lacking tdTomato+ (red) fibers but showing GFP+ (green) fibers (white arrows) innervating outer muscle (m) layers of a blood vessel. In some images, lumens are denoted by “L.” Scale bars denote 500 μm (***D***), 300 μm (***B***), 200 μm (***L***), 100 μm (***C***, ***I***, ***M***), 50 μm (***E***, ***F***, ***G***, ***J***, ***K***), 30 μm (***H***, ***N***), 20 μm (***P***), or 10 μm (***O***).

Pirt is expressed in almost all vagal (nodose and jugular) ganglion and DRG neurons (Patel et al., 2011; Kim et al., 2020b). Here, we investigated the overall sensory innervation of the lungs using four approaches: the Pirt-Ai9 (Fig. 2*A*), expressing tdTomato in all pirt-expressing cells; the Pirt-AAV-tdT_Lung_ (Fig. 3*A*), expressing tdTomato in lung afferents expressing Pirt; the Pirt-AAV-GFP_Vagal_ (Fig. 4*A*), expressing GFP in vagal afferents expressing pirt; and the Pirt-AAV-tdT_DRG_ (Fig. 4*A*), expressing tdTomato in DRG afferents expressing pirt.

In lung slices of Pirt-Ai9 (*n* = 8 animals, *n* = 16 lung slices), tdTomato+ fibers were found across multiple structures (Fig. 1*D*). Surprisingly, a small subset of airway smooth muscle cells also expressed tdTomato (Fig. 2*B*), which to a certain extent obscured the visualization of the tdTomato+ fibers within the airways. A total of 210 of the 219 conducting airways (96%) had tdTomato+ fibers (Fig. 1*D*,*E*), and these were found in a complicated plexus of fibers and identified terminations (Fig. 2*C*,*D*) beneath the epithelium. Typically, individual fibers were found just beneath the epithelium, whereas bundles of multiple fibers were found on the outside surface of the encircling smooth muscle cells (Fig. 2*E*). A total of 71 of the 219 conducting airways (32%) had tdTomato+ fibers that projected into the alveolar region, but this only occurred for airways <375 μm in diameter (Figs. 1*D*,*F*, 2*F*). A total of 86 of the 270 blood vessels (32%) had tdTomato+ fibers and this innervation was much less prevalent for small blood vessels (Figs. 1*D*,*H*, 2*G*,*H*). For some mice (*n* = 3 animals, *n* = 6 lung slices), we also injected colored latex beads into the pulmonary artery (pink) and pulmonary vein (blue) before dissection to selectively label these different vessels (Fig. 2*I–L*). Almost all vessels >25 μm contained either pink or blue latex, consistent with the lack of bronchial circulation in the mouse intrapulmonary airways (Mitzner et al., 2000). Consistent with other reports, pulmonary arteries invade the lung in conjunction with the bronchopulmonary tree, whereas pulmonary veins were often isolated. A total of 45 of the 101 pulmonary arteries (45%) had tdTomato+ fibers, and arteries without innervation were smaller than those with innervation (Mann–Whitney two-tailed *U* test, *p* = 0.0045; Fig. 2*M*). Only 26 of the 104 pulmonary veins (25%) had tdTomato+ fibers, but again there was a considerable correlation of innervation and diameter (Mann–Whitney two-tailed *U* test, *p* < 0.0001), with all large veins having Pirt+ innervation but only 9% of small vessels having Pirt+ innervation (Fig. 2*N*). tdTomato+ fibers were observed to penetrate the muscle layer surrounding the innervated arteries and veins. Some mouse intrapulmonary veins have a variable and discontinuous cardiomyocyte coat in close proximity to the vascular smooth muscle layer (Mueller-Hoecker et al., 2008), but these cells were not investigated in the current study.

To better visualize the Pirt+ innervation of the lung without the obscuring muscle cell tdTomato expression observed in Pirt-Ai9, we instilled rAAV2-flex-tdTomato into the lungs of *Pirt^Cre^* mice (*n* = 3 animals, *n* = 7 lung slices; Fig. 3*A*). Within the vagal ganglia, AAV-mediated expression of tdTomato was noted in 113 out of 3500 vagal neurons (3.2%; Fig. 3*B*). These airway-specific vagal neurons projected tdTomato+ central terminations to the brainstem nTS (and the neighboring area postrema; Fig. 3*C–F*), but no tdTomato+ fibers were found within the paratrigeminal complex (Pa5; Fig. 3*C*,*D*). Interestingly, we found no tdTomato+ neurons within the DRG (out of 4522 neurons), indicating that these neurons were not labeled by lung instillation with rAAV2. In the lung, 107 of the 127 conducting airways (84%) had tdTomato+ fibers (Figs. 1*D*,*E*, 3*G*). Almost no tdTomato+ fibers penetrated the airway epithelium of conducting airways (Fig. 3*H*). Instead tdTomato+ fibers were found within the lamina propria and smooth muscle layers, as well as on the outside surface of the smooth muscle (Fig. 3*H*). The tdTomato+ fibers within the lamina propria appeared thinner than the fibers on the surface of the smooth muscle, but tracing indicated that these were the same fiber populations, which got progressively thinner when they penetrated the smooth muscle layer. Confirmed tdTomato+ terminations were also occasionally found within the conducting airways (Fig. 3*I*). A total of 44 of the 127 conducting airways (35%) had tdTomato+ fibers that projected into the alveolar region, but again this only occurred for airways <375 μm in diameter (Figs. 1*D*,*F*, 3*J*). Only 17 of the 134 of blood vessels (13%) had tdTomato+ fibers and, unlike the Pirt-Ai9 dataset, none of the large blood vessels were innervated (Figs. 1*D*,*H*, 3*K–M*). For blood vessels <375 μm in diameter, the tdTomato+ fibers appeared to innervate multiple layers of the muscle, in some cases penetrating close to the endothelial layer (Fig. 3*K–M*).

The simultaneous injection of AAV9-flex-GFP into the vagal ganglia and AAV9-flex-tdTomato into the thoracic DRG of Pirt^Cre^ mice (*n* = 3 animals, *n* = 7 lung slices; Fig. 4*A*) produced robust and selective reporter expression in neurons within these ganglia (Fig. 4*B*,*C*). In the lung, 194 of the 229 conducting airways (85%) had GFP+ fibers (vagal), whereas only 46/229 (20%) had tdTomato+ fibers (DRG; Figs. 1*D*, 4*D–L*). There was a considerable correlation of airway diameter and DRG Pirt+ innervation, with tdTomato+ fibers in 100% and 12% of large and small airways, respectively (Figs. 1*E*, 4*D*). The GFP+ and tdTomato+ fibers were part of the same loose plexus surround the conducting airways, beneath the epithelium (Fig. 4*H*). A total of 114 of the 229 conducting airways (50%) had GFP+ fibers (vagal) that projected out into the alveolar region, whereas none of the tdTomato+ fibers (DRG) projected into the alveolar region (Figs. 1*D*,*F*, 4*D–L*). A total of 31 of the 131 blood vessels (24%) had GFP+ fibers (vagal) compared with just 10/131 (8%) with tdTomato+ fibers (DRG; Figs. 1*D*, 4*M–P*). Although both innervations were more prevalent for larger blood vessels, this correlation was much more extreme for DRG Pirt+ fibers which almost exclusively innervated only large blood vessels (Fig. 1*H*). Qualitative analysis of the blood vessel innervation suggested that GFP+ fibers (vagal) innervate the outer muscle layers, whereas tdTomato+ fibers (DRG) sometimes project through the muscle layers to come in close apposition to the endothelial layer (Fig. 4*M–P*).

### 5HT_3_^Cre^

**Figure 5.**
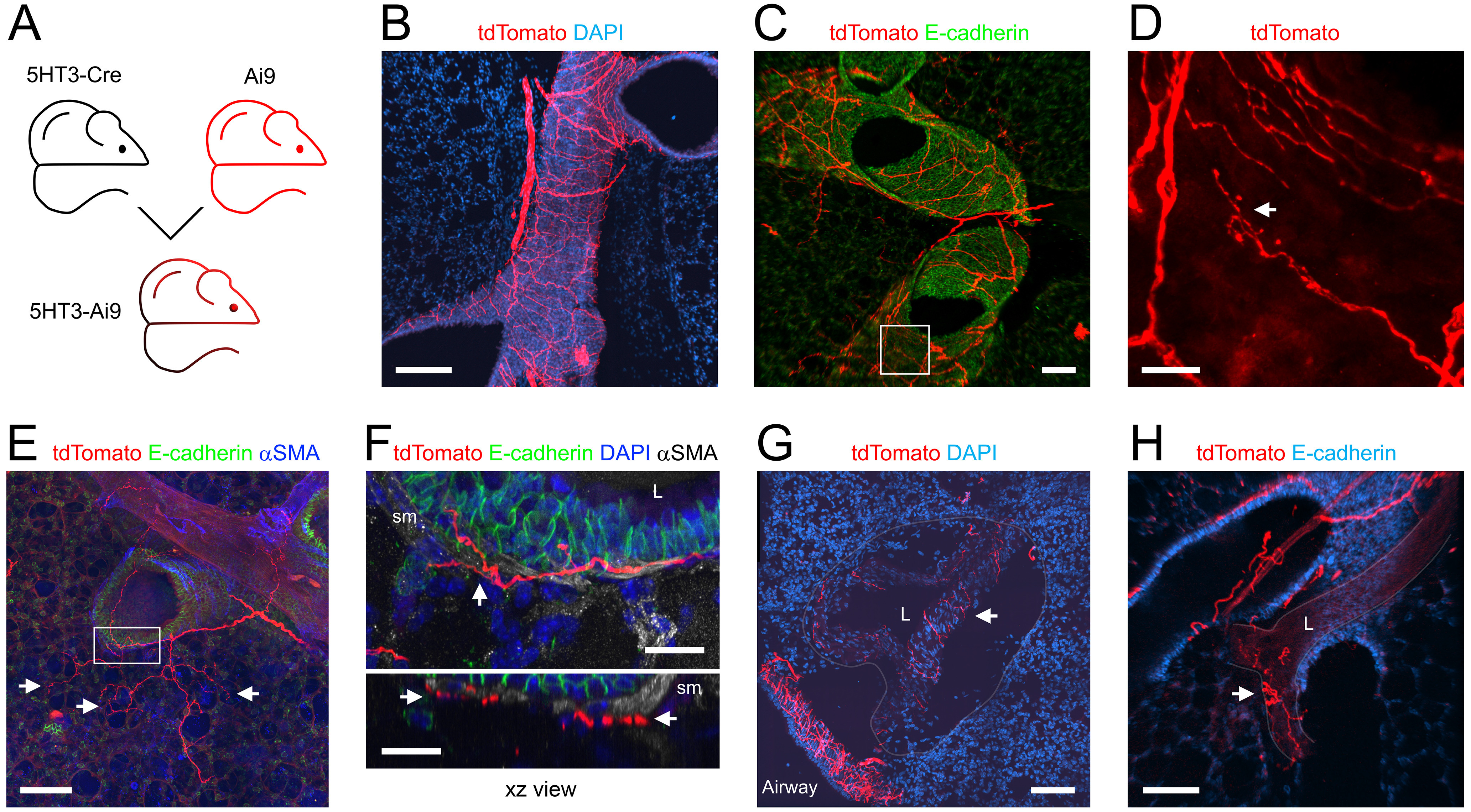
Mapping the lung innervation by 5HT_3_+ nerves. ***A***, Approach for labeling all 5HT_3_+ afferents with tdTomato. ***B***, Lung slice stained for DAPI (blue) showing tdTomato-expressing (red) nerves innervating a large conducting airway. ***C***, Lung slice stained for E-Cadherin (green) showing tdTomato-expressing nerves (red) innervating conducting airways. ***D***, Higher magnification of white box in ***C*** (red channel only), with identified tdTomato+ nerve terminals (white arrows). ***E***, Lung slice stained for α smooth muscle actin (αSMA; blue) and E-Cadherin (green) showing tdTomato+ (red) fibers projecting from a conducting airway into the alveolar region (identified terminals denoted by white arrows). ***F***, Higher magnification of white box in ***D*** (E-cadherin in green, αSMA in white, DAPI in blue), showing tdTomato+ fibers (red) intercalated (white arrows) with the smooth muscle (sm) layers surrounding the conducting airway. ***G***, ***H***, Lung slice stained for DAPI (blue) showing tdTomato-expressing (red) nerves innervating blood vessels (white arrows). Vessel outlines have been superimposed by gray lines. In some images, lumens are denoted by “L.” Scale bars denote 200 μm (***B***), 100 μm (***C***, ***E***, ***G***, ***H***), or 20 μm (***D***, ***F***).

The serotonergic 5HT_3_ receptor is widely expressed in the nodose ganglion but is rarely expressed in jugular and DRG neurons (Chuaychoo et al., 2005; Usoskin et al., 2015; Kim et al., 2020b). In the lungs of 5HT_3_-Ai9 (*n* = 7 animals, *n* = 19 lung slices; Fig. 5*A*), tdTomato expression was almost exclusively found in nerve fibers (Fig. 5*B–H*). A total of 257 of the 294 conducting airways (84%) had tdTomato+ fibers (Figs. 1*D*,*E*, 5*B–F*). Confirmed terminations of tdTomato+ fibers were found in the plexus surrounding the conducting airways (Fig. 5*D*). A total of 122 of the 294 conducting airways (41%) had tdTomato+ fibers that projected into the alveolar region (Figs. 1*D*,*F*, 5*E*). Similar to the Pirt+ fibers, 5HT_3_+ fibers innervating the conducting airways intercalated with the smooth muscle layer and lamina propria (Fig. 5*F*). Only 30 of the 211 blood vessels (14%) had tdTomato+ fibers (Figs. 1*D*,*H*, 5*G*,*H*). In particular, fewer large diameter blood vessels had tdTomato+ innervation in the 5HT_3_-Ai9 (32%) compared with Pirt-Ai9 (85%; Fig. 1*H*).

### Tac1^Cre^

**Figure 6.**
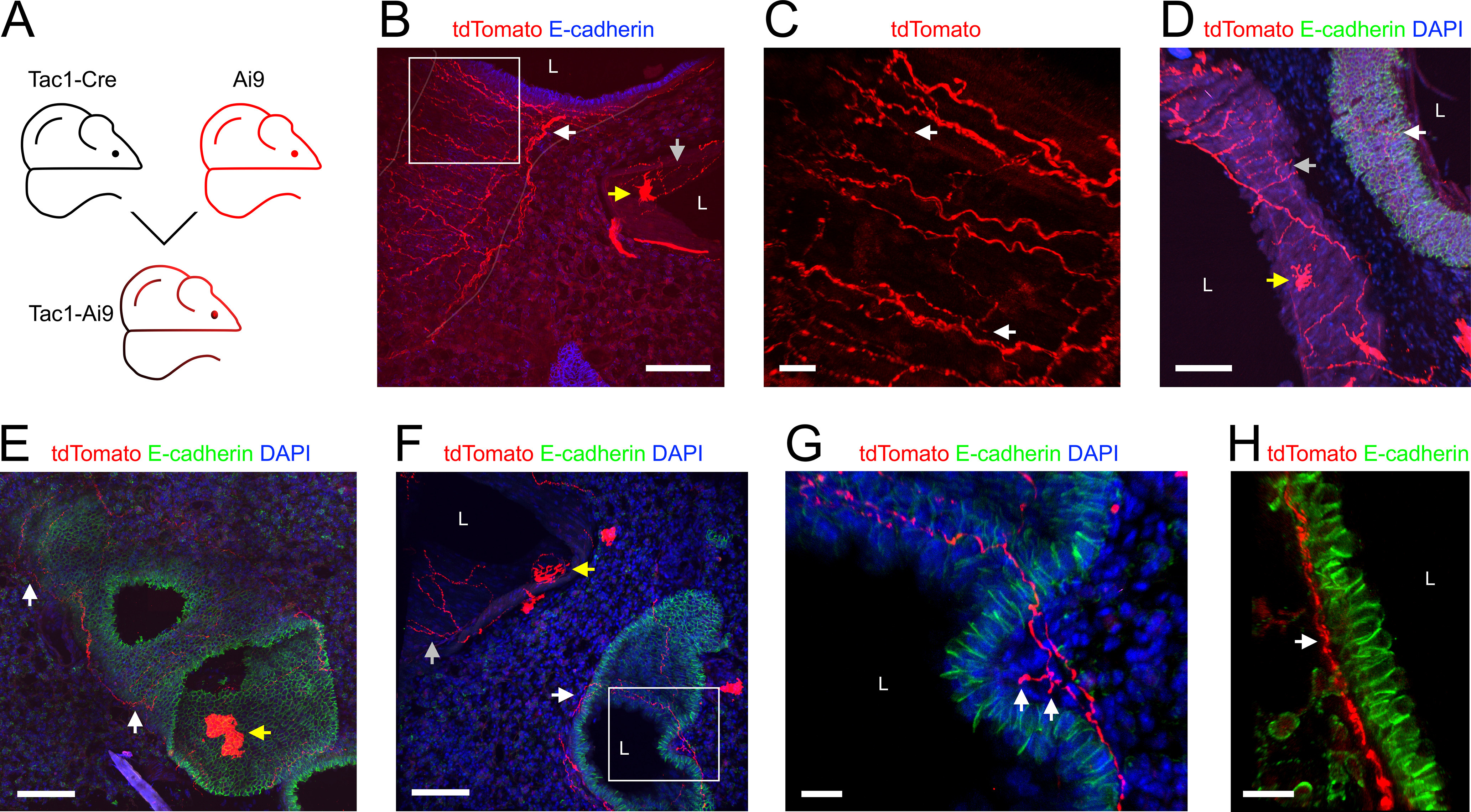
Mapping the lung innervation by Tac1+ nerves. ***A***, Approach for labeling all Tac1+ afferents with tdTomato. ***B***, Lung slice stained for E-cadherin (blue) showing tdTomato-expressing (red) nerves innervating a large conducting airway (white arrow) and a blood vessel (gray arrow). The trench of the conducting airway (outline identified by superimposed white line) cuts through the slice from top to bottom left. Note the presence of tdTomato+ cells within the blood vessel wall (yellow arrow). ***C***, Higher magnification of white box in ***B*** (red channel only), with identified tdTomato+ nerve terminals (white arrows). ***D–F***, Lung slice stained for DAPI (blue) and E-Cadherin (green) showing tdTomato+ (red) fibers innervating conducting airways (white arrows) and blood vessels (gray arrows). tdTomato+ cells are also present (yellow arrows). ***G***, Higher magnification of white box in ***F*** showing an identified tdTomato-expressing (red) nerve terminal (white arrows) innervating a conducting airway. ***H***, Lung slice stained for E-Cadherin (green) showing tdTomato-expressing (red) nerves just beneath the epithelial layer of a conducting airway (white arrow). In some images, lumens are denoted by “L.” Scale bars denote 100 μm (***B***, ***D***–***F***) or 20 μm (***C***, ***G***, ***H***).

**Figure 7.**
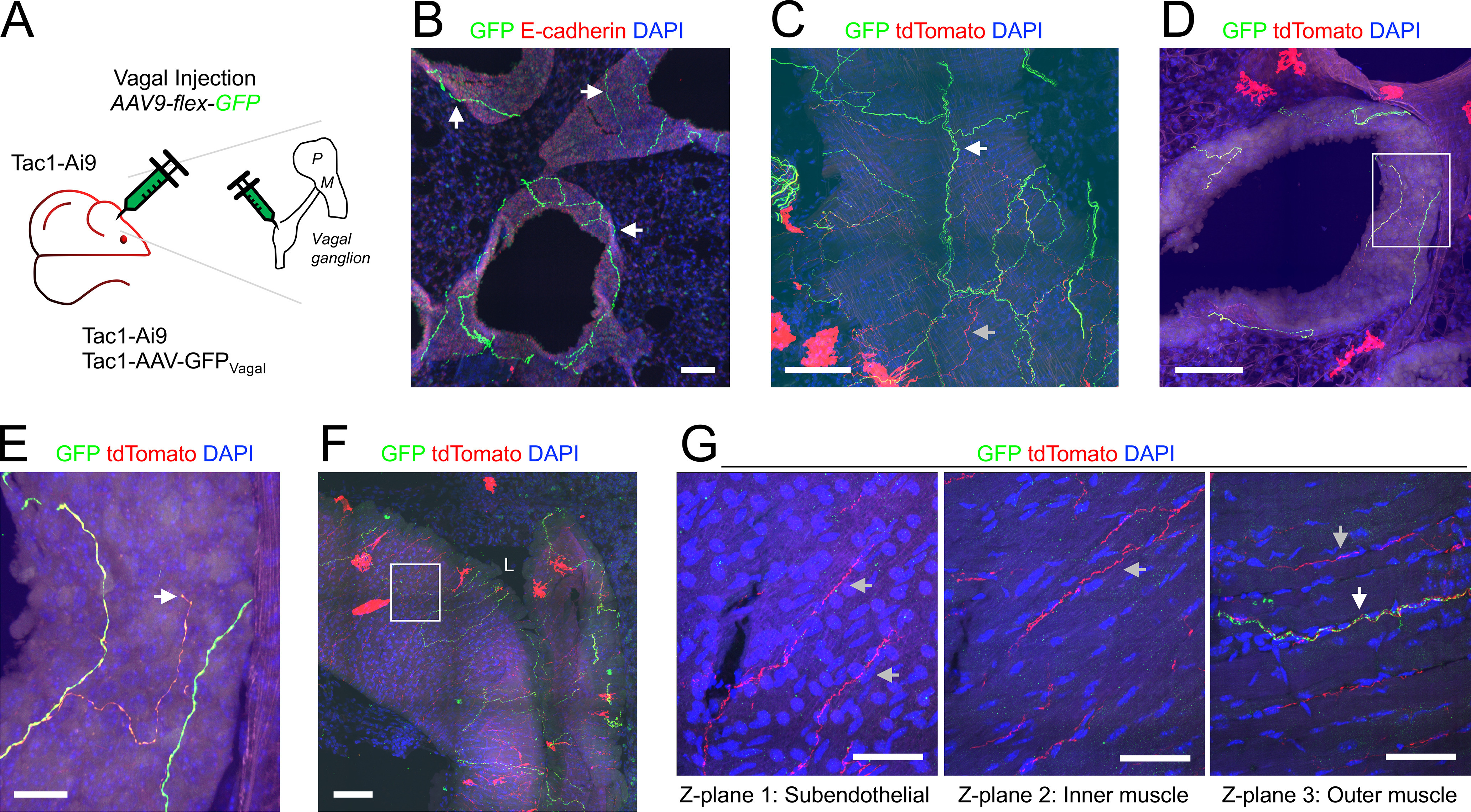
Mapping the lung innervation by vagal Tac1+ nerves. ***A***, Approach for labeling all Tac1+ afferents with tdTomato and vagal Tac1+ afferents with GFP. ***B***, Lung slice stained for E-cadherin (red) and DAPI (blue) showing GFP-expressing (green) nerves innervating conducting airways (white arrows). ***C***, Lung slice stained for DAPI (blue) showing a large conducting airway trench innervated by GFP-expressing (green) nerves (white arrow) and tdTomato-expressing (red) nerves (gray arrow). ***D***, Lung slice stained for DAPI (blue) showing a conducting airway innervated by fibers expressing both GFP (green) and tdTomato (red). ***E***, Higher magnification of white box in ***D***, with identified tdTomato+ nerve terminal (white arrow). ***F***, Lung slice stained for DAPI (blue) showing a large blood vessel (slightly folded) innervated by GFP-expressing (green) nerves and tdTomato-expressing (red) nerves. ***G***, Individual *z*-planes (1–3) of the white box in ***F***, at higher magnification, showing fibers expressing only tdTomato innervating the inner muscle layers (gray arrows), whereas GFP-expressing fibers (white arrow) innervate only the outer muscle layer. In some images, lumens are denoted by “L.” Scale bars denote 100 μm (***B***, ***C***, ***D***, ***F***), 50 μm (***G***), or 20 μm (***E***).

**Figure 8.**
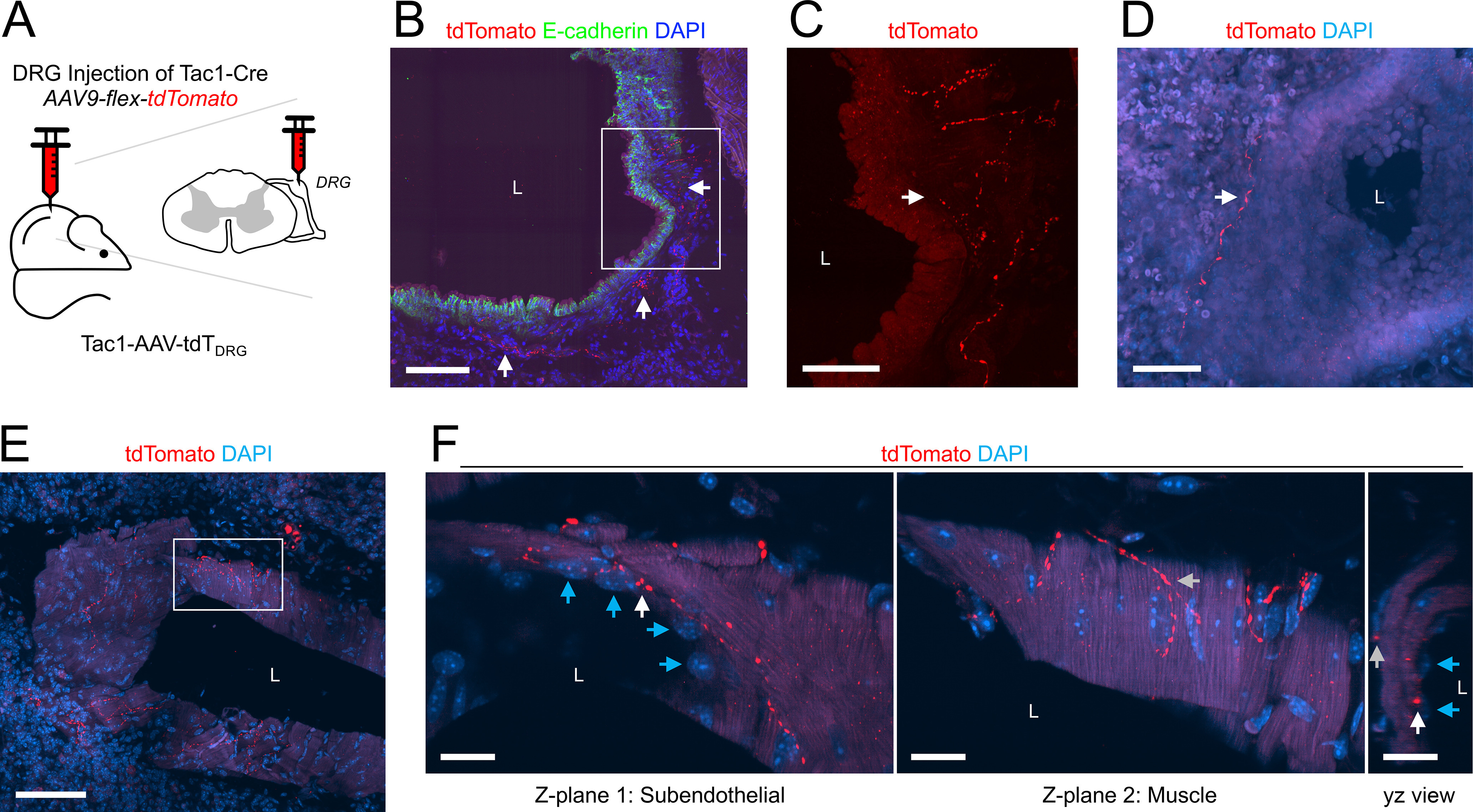
Mapping the lung innervation by Tac1+ fibers projected from DRG. ***A***, Approach for labeling Tac1+ neurons in the DRG with tdTomato. ***B***, Lung slice stained for E-cadherin (green) and DAPI (blue) showing tdTomato+ (red) fibers innervating a large conducting airway (white arrows). ***C***, Higher magnification of white box in ***B*** (red channel only) showing tdTomato+ fibers (white arrow). ***D***, ***E***, Lung slice stained for DAPI (blue) showing tdTomato+ (red) fibers innervating a small conducting airway (white arrow, ***D***) and a large blood vessel (***E***). ***F***, Individual *z*-planes (1, 2, left) and the corresponding *yz* view (right) of the white box in ***E***, at higher magnification, with identified endothelial cells (blue arrows) adjacent to the vessel lumen, showing fibers expressing tdTomato innervating both the subendothelial layer (white arrows) and the muscle layers (gray arrows). In some images, lumens are denoted by “L.” Scale bars denote 100 μm (***B***, ***E***), 50 μm (***C***, ***D***), or 20 μm (***F***).

Tac1, the gene for preprotachykinin (precursor for the tachykinin neuropeptide substance P), is expressed in jugular and DRG neurons, but is expressed in very few nodose neurons innervating the airways (Ricco et al., 1996; Undem et al., 2004; Nassenstein et al., 2010; Usoskin et al., 2015; Kim et al., 2020b). Here, we investigated Tac1+ innervation of the lungs using three approaches: Tac1-Ai9 (Fig. 6*A*), expressing tdTomato in all Tac1-expressing cells; the Tac1-AAV-GFP_Vagal_ (Fig. 7*A*), expressing GFP in vagal afferents expressing Tac1; and the Tac1-AAV-tdT_DRG_ (Fig. 8*A*), expressing tdTomato in DRG afferents expressing Tac1. In the lungs of Tac1-Ai9 mice (*n* = 11 animals, *n* = 27 lung slices), tdTomato+ fibers were noted in both conducting airways and blood vessels (Fig. 6*B*). In addition, tdTomato was expressed by a subset of intrinsic cells found in the vasculature and occasionally the conducting airways. A total of 228 of the 281 conducting airways (81%) had tdTomato+ fibers (Figs. 1*D*,*E*, 6*B–H*). Smaller conducting airways were less likely to have Tac1+ innervation (67%) compared with medium and large airways (96% and 100%, respectively; Fig. 1*E*). tdTomato+ fibers and their confirmed terminations were noted in the plexus beneath the epithelial layer of the conducting airways (Fig. 6*B–H*). Strikingly, no tdTomato+ fibers in the Tac1-Ai9 mouse lung projected into the alveolar region (Figs. 1*D*,*F*, 6*B–H*). 59 of the 259 blood vessels (23%) had tdTomato+ fibers (Figs. 1*D*,*H*, 6*B*,*D*). Tac1+ innervation was particularly prevalent in large blood vessels (80%; Fig. 1*H*).

Some of the Tac1-Ai9 mice received an AAV9-flex-GFP injection into their vagal ganglia to compare the overall Tac1+ innervation (tdTomato) with the vagal-specific Tac1+ innervation (GFP; *n* = 5 animals, *n* = 14 lung slices; Fig. 7*A*). GFP+ terminations were found in both conducting airways and blood vessels (Fig. 7*B–G*). As expected, all GFP+ fibers also expressed tdTomato, but some tdTomato+ fibers lacked GFP (Fig. 7*C*,*F*,*G*), suggesting these particular Tac1+ fibers were not projected from the vagal ganglia (possibly projected from DRG). A total of 162 of the 208 conducting airways (78%) had GFP+ fibers within the plexus surrounding the conducting airways (Figs. 1*D*,*E*, 7*B–E*). tdTomato+/GFP-negative fibers were more common in large conducting airways compared with smaller airways. Confirmed vagal Tac1+ terminations were noted (Fig. 7*E*). No GFP+ fibers projected from the conducting airways into the alveolar region (Figs. 1*D*,*F*, 7*B–E*). A total of 34 of the 188 blood vessels (18%) had GFP+ fibers, and like the tdTomato+ (overall) Tac1+ innervation, this was more common in large blood vessels. Comparison of Tac1+ fibers innervating large blood vessels indicated that the confirmed vagal Tac1+ fibers (tdTomato+/GFP+) innervated only the outer muscle layers, whereas the presumed nonvagal Tac1+ fibers (tdTomato+/GFP-negative) projected through the muscle layers to within close proximity with the endothelial layer (Fig. 7*G*).

A lack of GFP expression in Tac1+ fibers following vagal injection with AAV9-flex-GFP is not definitive evidence that the fiber does not project from vagal neurons (as transfection is rarely 100%). As such, we investigated the DRG Tac1+ innervation by injecting the thoracic DRG of Tac1^Cre^ mice with AAV9-flex-tdTomato (*n* = 3 animals, *n* = 5 lung slices; Fig. 8*A*). Only 12 of the 153 conducting airways (8%) had tdTomato+ fibers (Figs. 1*D*, 8*B–D*). DRG Tac1+ innervation was almost exclusively restricted to the large airways, of which 71% had tdTomato+ fibers (Fig. 1*E*). As expected, no tdTomato+ fibers projected into the alveolar region. Only eight of the 118 blood vessels (7%) had tdTomato+ fibers (Figs. 1*D*, 8*E*,*F*) and this innervation was restricted to medium and large blood vessels. In some cases, DRG Tac1+ fibers projected through the muscle layers to come into close contact with the endothelial cells (Fig. 8*F*).

### TRPV1^Cre^

**Figure 9.**
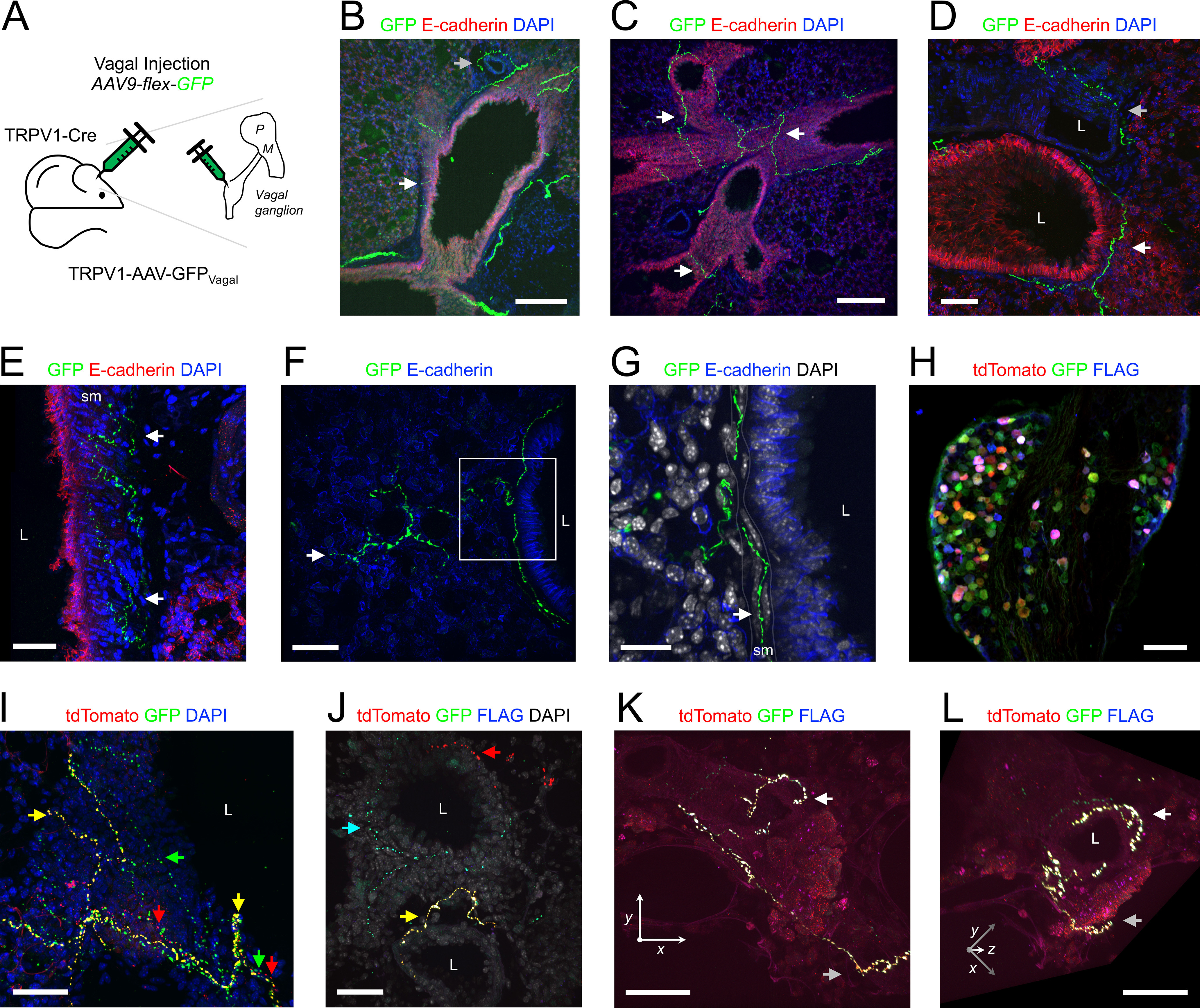
Mapping the lung innervation by vagal TRPV1+ nerves. ***A***, approach for labeling vagal TRPV1+ afferents with GFP. ***B–E***, lung slice stained for E-cadherin (red) and DAPI (blue) showing GFP-expressing (green) nerves innervating conducting airways (white arrows) and blood vessels (gray arrows). Note that GFP+ fibers are found within the smooth muscle layer of the large conducting airways in ***E***. ***F***, Lung slice stained for E-Cadherin (blue) showing a GFP-expressing (green) nerve projecting from a small conducting airway into the alveolar region (white arrow denotes identified terminal). ***G***, Higher magnification of white box in ***F***, with DAPI (white), showing GFP+ fiber intercalating (white arrow) with smooth muscle (sm) cells surrounding the conducting airway. ***H***, Vagal ganglia of TRPV1^Cre^ mouse following vagal injection of triple cocktail of AAV9-flex-reporters: tdTomato (red), GFP (green), and FLAG (blue). ***I–L***, lung slice of mouse from ***H***. ***I***, Lung slice stained for DAPI (blue) showing multiple individual fibers innervating large conducting airway including GFP+ fibers (green arrow), GFP+/tdTomato+ fibers (yellow arrows), and tdTomato+ fibers (red arrows). ***J***, Lung slice stained for DAPI (white) showing individual GFP+ (green), tdTomato+ (red), and FLAG+ (blue) fibers innervating small conducting airways (red arrow, cyan arrow and yellow arrow). ***K***, Lung slice showing a single GFP+/tdTomato+/FLAG+ fiber innervating a small blood vessel (identified terminal denoted by white arrow, parental axon denoted by gray arrow). ***L***, 3-dimensional rotation of K to visualize the circumferential structure of the TRPV1+ terminal innervating the vessel. In some images, lumens are denoted by “L.” Scale bars denote 200 μm (***B***, ***C***), 100 μm (***H***), 50 μm (***D***, ***E***, ***F***, ***I***, ***J***, ***K***, ***L***), or 20 μm (***G***).

**Figure 10.**
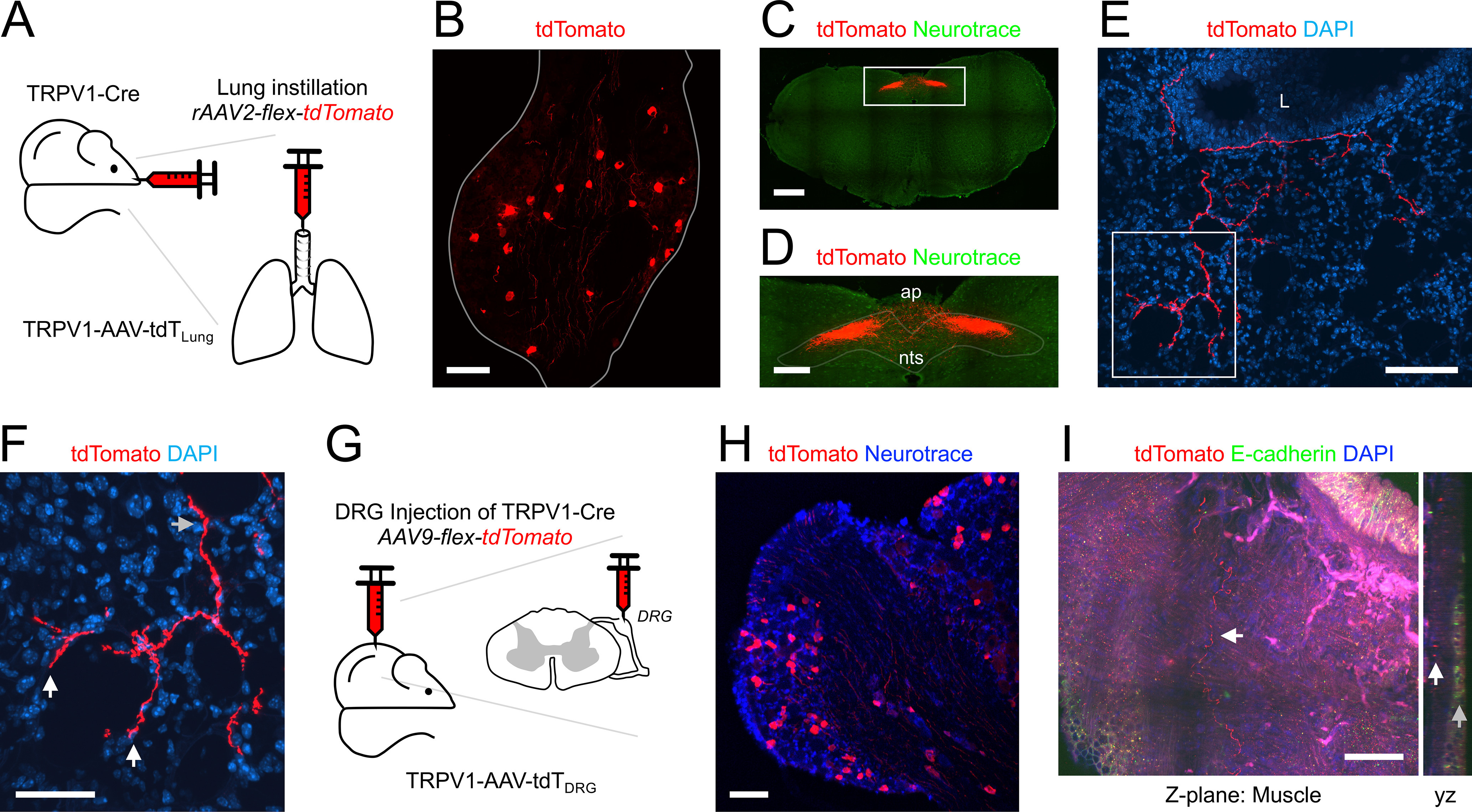
Mapping the lung innervation by TRPV1+ fibers labeled by lung instillation or by DRG injection of AAV vectors. ***A***, Approach for labeling TRPV1+ afferents innervating the lungs. ***B***, Vagal ganglia showing lung-labeled tdTomato+ (red) neurons. ***C***, Coronal slice of brainstem (600 μm caudal of obex) stained for Neurotrace (green) showing central projections of lung-labeled tdTomato+ (red) afferents. ***D***, Higher magnification of white box in ***C***, highlighting the nTS and area postrema (ap). ***E***, Lung slice stained for DAPI (blue) showing a tdTomato+ fiber (red) projecting from a small conducting airway into the alveolar region. Lumen is denoted by “L.” ***F***, Higher magnification of the white box in ***E***, showing identified nerve terminals (white arrows) and parental axon (gray arrow). ***G***, Approach for labeling TRPV1+ neurons in the DRG with tdTomato. ***H***, DRG showing tdTomato+ (red) neurons. ***I***, Lung slice stained for E-Cadherin (green) and DAPI (blue) showing (left) the muscle layer of a trench of a large conducting airway and (right) the *yz* view (epithelial layer identified by gray arrow). Note the single tdTomato+ (red) fiber innervating the smooth muscle layer (white arrow). Scale bars denote 500 μm (***C***), 200 μm (***D***), 100 μm (***B***, ***E***, ***H***), or 50 μm (***F***, ***I***).

TRPV1 is a well-characterized marker of nociceptive sensory nerves in both the vagal ganglia and the DRG (Undem et al., 2004; Nassenstein et al., 2010; Usoskin et al., 2015; Wang et al., 2017; Kupari et al., 2019; Mazzone et al., 2020; Kim et al., 2020b). We studied TRPV1+ lung innervation using three approaches: TRPV1-AAV-GFP_Vagal_ (Fig. 9*A*), expressing GFP in vagal afferents expressing TRPV1; TRPV1-AAV-tdT_Lung_ (Fig. 10*A*), expressing tdTomato in lung afferents expressing TRPV1; and TRPV1-AAV-tdT_DRG_ (Fig. 10*G*), expressing tdTomato in DRG afferents expressing TRPV1. In the lungs of TRPV1^Cre^ mice injected with AAV9-flex-GFP into their vagal ganglia (*n* = 10 animals, *n* = 19 lung slices), we found GFP+ fibers innervating 235 of the 313 conducting airways (75%; Figs. 1*D*, 9*B–G*). More than 95% of medium and large airways had vagal TRPV1+ innervation compared with 69% of small airways (Figs. 1*E*, 9*C*). GFP+ fibers were found within the smooth muscle layer of the conducting airways (Fig. 9*B–G*). A total of 89 of the 313 conducting airways (28%) had GFP+ fibers that projected into the alveolar region, and again this only occurred for small and medium airways (Figs. 1*D*,*F*, 9*F*). A total of 77 of the 288 blood vessels (27%) had GFP+ fibers (Figs. 1*D*, 9*D*), but there did not seem to be a substantial difference between vessels of different diameters (Fig. 1*H*). In three TRPV1^Cre^ mice (*n* = 6 lung slices), we injected a triple cocktail of AAV9-flex-tdTomato, -GFP, and -Flag into the vagal ganglia, to distinguish individual TRPV1+ neurons/fibers based on random distribution of reporters (Fig. 9*H*). In the lungs of these mice, we often found multiple TRPV1+ fibers innervating individual conducting airways (Fig. 9*I*,*J*). On the other hand, blood vessels, particularly smaller vessels, tended to be innervated by single TRPV1+ fibers (Fig. 9*K*,*L*).

Instillation of rAAV2-flex-tdTomato into the lungs of TRPV1^Cre^ mice (*n* = 4 animals, *n* = 9 lung slices; Fig. 10*A*) resulted in AAV-mediated expression of tdTomato in 113 out of 3298 vagal neurons (3.4%) within the vagal ganglia (Fig. 10*B*). These airway-specific vagal TRPV1+ neurons projected tdTomato+ central terminations to the brainstem nTS and neighboring area postrema (Fig. 10*C*,*D*), but not to the Pa5 (Fig. 10*C*). Based on our Pirt^Cre^ studies, we did not assess tdTomato expression in the DRG of these mice. In the lungs, 133 of the 161 conducting airways (83%) had tdTomato+ fibers (Figs. 1*D*,*E*, 10*E*). Furthermore, 70 of the 161 conducting airways (43%) had tdTomato+ fibers that projected into the alveolar region (Figs. 1*D*,*F*, 10*E*,*F*). Once again this only occurred for small and medium diameter airways (Fig. 1*F*). Only nine of the 139 blood vessels (6%) had tdTomato+ fibers, and such TRPV1+ innervation was completely absent from large vessels (Fig. 1*D*,*H*).

Injecion of AAV9-flex-tdTomato into the thoracic DRG of TRPV1^Cre^ mice (*n* = 3 animals, *n* = 3 lung slices; Fig. 10*G*) produced robust tdTomato expression in a subset of DRG neurons (Fig. 10*H*). Nevertheless, very few structures within the lungs of these animals were innervated by tdTomato+ fibers. Only two of the 82 conducting airways (2%) had DRG TRPV1+ innervation, and this was exclusively restricted to large airways (Figs. 1*D*,*E*, 10*I*). The very sparse tdTomato+ fibers were found within the airway smooth muscle layer (Fig. 10*I*). Similarly, only one of the 57 blood vessels (2%) had tdTomato+ fibers, and this too was exclusively restricted to large blood vessels (Fig. 1*D*,*H*).

### CGRP

**Figure 11.**
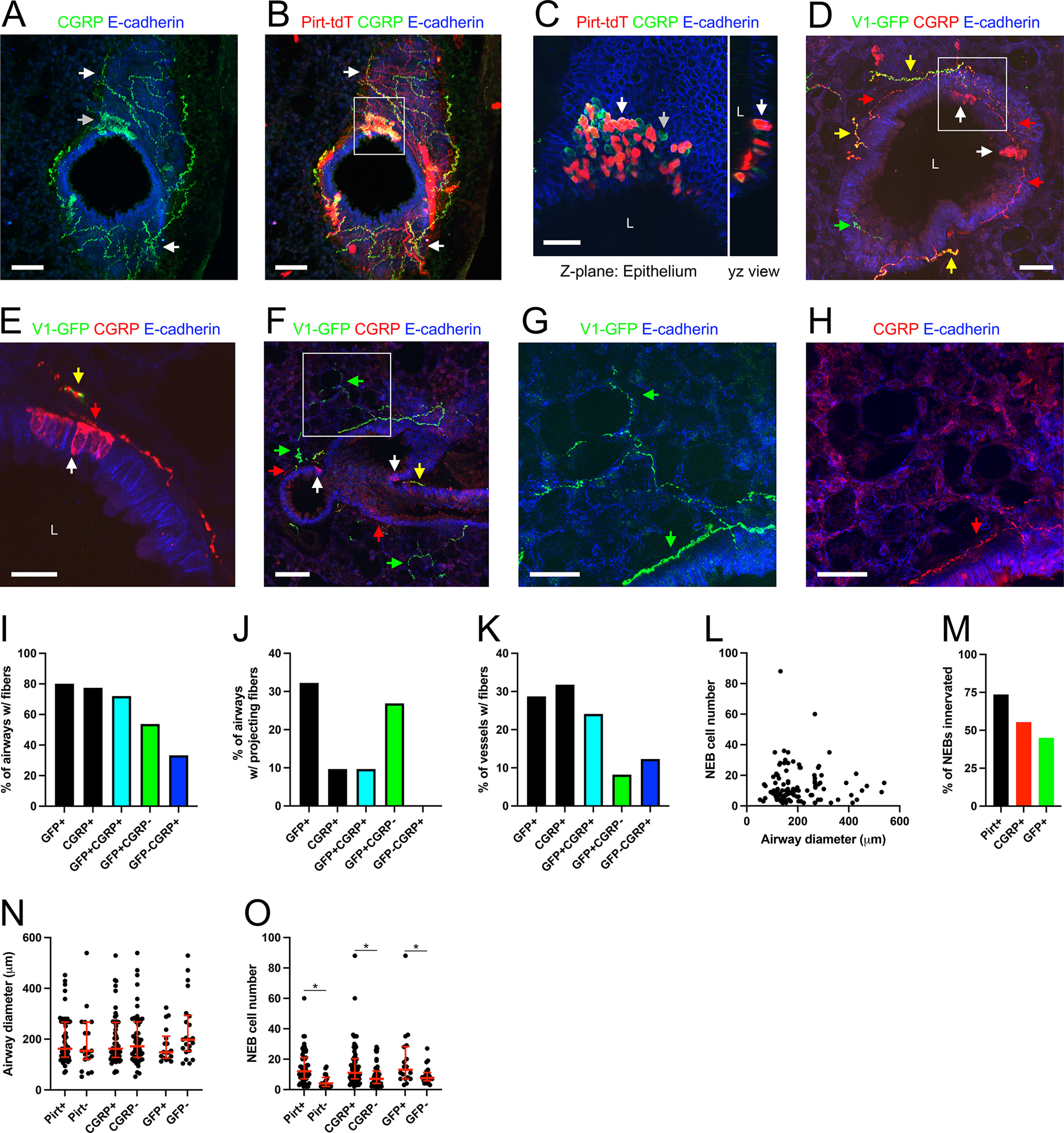
Mapping the lung innervation by CGRP. ***A–C***, Pirt-Ai9 lung slice stained for CGRP (green), E-Cadherin (blue), and tdTomato+ pirt-expressing fibers/cells (red, in ***B***, ***C***). ***A***, ***B***, CGRP+ fibers (white arrows) innervating a conducting airway. Note the cluster of CGRP+ epithelial cells (gray arrow) comprising an NEB. ***C***, Higher magnification of white box in ***B***, showing the NEB with both CGRP+/Pirt+ cells (white arrow) and CGRP+/Pirt-negative cells (gray arrow). Note the gain of the red and green channels have been decreased to better visualize the NEB cells. Thus, less bright CGRP+ and Pirt+ fibers are no longer visible. ***D–H***, lung slice of TRPV1^Cre^ mouse following vagal injection with AAV9-flex-GFP, stained for CGRP (red), E-Cadherin (blue), and GFP+ TRPV1-expressing vagal fibers (green). ***D***, ***F***, GFP+/CGRP+ (yellow arrows), GFP+/CGRP-negative (green arrows) and GFP-negative/CGRP+ (red arrows) fibers innervate the conducting airways. Also shown are CGRP+ NEBs (white arrows). ***E***, Higher magnification of white box in ***D***, showing an NEB with associated innervation by both GFP+/CGRP+ (yellow arrows) and GFP-negative/CGRP+ (red arrows) fibers. ***G***, ***H***, Higher magnification of white box in ***F*** showing GFP+ fibers innervating a conducting airway and projecting into the alveolar region (green arrow, in ***G***) and CGRP+ fibers innervating a conducting airway but not projecting into the alveolar region (red arrow, in ***H***). ***I–K***, Quantification the % of conducting airways with fibers (***I***), the % of conducting airways with fibers that project out to the alveolar region (***J***), and the % of blood vessels with fibers (***K***) for CGRP+ fibers and TRPV1+ (GFP+) fibers. Data in ***I–K*** are derived from three TRPV1^Cre^ mice with AAV9-flex-GFP vagal injections (186 conducting airways, 195 blood vessels). ***L***, Correlation of the number of CGRP+ cells within an NEB and the diameter of the conducting airway. ***M***, Percentage of NEBs innervated by Pirt+, CGRP+, and TRPV1+ (GFP+) fibers. ***N***, Median (with interquartile range) airway diameter of conducting airways at locations of individual NEBs, grouped by innervation (or lack of) by Pirt+, CGRP+, and TRPV1+ (GFP+) fibers. ***O***, Median (with interquartile range) number of CGRP+ cells within the NEBs, grouped by innervation (or lack of) by Pirt+, CGRP+, and TRPV1+ (GFP+) fibers. Data in ***L–O*** are derived from three TRPV1^Cre^ mice with AAV9-flex-GFP vagal injections and three Pirt-Ai9 mice (in total, 112 NEBs). * denotes significant difference between NEBs with and without innervation (Mann–Whitney two-tailed *U* test, *p* < 0.05, see text for precise values). In some images, lumens are denoted by “L.” Scale bars denote 100 μm (***A***, ***B***, ***F***), 50 μm (***D***, ***G***, ***H***), 30 μm (***C***), or 20 μm (***E***).

CGRP is a neuropeptide expressed in nociceptive subsets of vagal and DRG neurons (Springall et al., 1987; Helke and Hill, 1988; Zhuo et al., 1997; Usoskin et al., 2015; Wang et al., 2017; Kupari et al., 2019; Mazzone et al., 2020). Although CGRP and substance P expression largely overlap, CGRP expression is more prevalent in nodose neurons than substance P/Tac1. CGRP was determined using IHC in lung slices of Pirt-Ai9 mice (*n* = 3 animals, *n* = 4 lung slices) and TRPV1^Cre^ mice following vagal injection with AAV9-flex-GFP (*n* = 3 animals, *n* = 5 lung slices). In total (*n* = 6 animals, *n* = 9 lung slices), 228 of the 296 conducting airways (77%) had CGRP+ fibers (Figs. 1*D*,*E*, 11). Interestingly, 30 of the 296 conducting airways (10%) had CGRP+ fibers that projected into the alveolar region (Figs. 1*D*,*F*, 11). A total of 92 of the 279 blood vessels (33%) had CGRP+ innervation (Figs. 1*D*,*H*, 11). Analysis of the Pirt-Ai9 data alone indicated that some tdTomato+ fibers (i.e., Pirt+) expressed CGRP (Fig. 11*B*). As expected, there were no CGRP+ fibers that lacked Pirt expression, indicating that this neuropeptide was expressed in afferent nerves but not efferent nerves. Analysis of the TRPV1^Cre^ mice following vagal injection with AAV9-flex-GFP data alone indicated that the 186 conducting airways were innervated by GFP+/CGRP+, GFP+/CGRP-negative, and GFP-negative/CGRP+ fibers (Fig. 11*D–I*). Interestingly, the predominant fiber type projecting from the conducting airways into the alveolar region was the GFP+/CGRP-negative subset (Fig. 11*F–H*,*J*). We did occasionally observe alveolar projecting fibers that were GFP+/CGRP+ but none were GFP-negative/CGRP+ (Fig. 11*F–H*,*J*). Whereas for the 195 blood vessels, the predominant fiber type was the GFP+/CGRP+ subset, with sporadic GFP+/CGRP-negative and GFP-negative/CGRP+ innervation (Fig. 11*K*).

In addition to CGRP expression in nerve fibers, CGRP was robustly expressed in clusters of epithelial cells termed NEBs (Fig. 11*A–F*). A total of 112 CGRP+ NEBs were found throughout the 296 conducting airways. The number of CGRP+ cells within a particular NEB varied from 2 to 88 (mean of 12.8 ± 1.1), solitary CGRP+ cells were excluded from this analysis. There did not appear to be any correlation between the number of cells within an NEB and the diameter of the conducting airway (Fig. 11*L*). In the lungs of Pirt-Ai9, 45 of the 74 CGRP+ NEBs (60%) unexpectedly contained cells that also expressed tdTomato (Fig. 11*B*,*C*), including all NEBs comprised of more than three CGRP+ cells. The mean number of tdTomato+ cells within an NEB was 5.7 ± 2.5. Of the 112 CGRP+ NEBs studied in total, 62 (55%) were innervated by CGRP+ fibers (Fig. 11*M*). In the lungs of Pirt-Ai9, 53 of the 72 CGRP+ NEBs (74%) were innervated by tdTomato+ fibers (Fig. 11*M*). NEBs were innervated by both CGRP+ and CGRP-negative fibers expressing tdTomato. In the lungs of TRPV1^Cre^ mice following vagal injection of AAV9-flex-GFP, 18 of the 40 (45%) CGRP+ NEBs were innervated by GFP+ fibers (Fig. 11*M*). Almost all the GFP+ fibers (i.e., vagal TRPV1+ fibers) innervating the NEBs also expressed CGRP. Importantly, multiple NEBs were innervated by GFP-negative/CGRP+ fibers (Fig. 11*D*,*E*). There were no differences in the diameters of conducting airways with NEBs that were or were not innervated by Pirt+, CGRP+, and GFP+ (vagal TRPV1+) fibers (Mann–Whitney two-tailed *U* test, *p* = 0.49, *p* = 0.74, and *p* = 0.09, respectively; Fig. 11*N*). Nevertheless, the number of CGRP+ cells within an innervated NEB was significantly greater than the number within noninnervated NEBs (Mann–Whitney two-tailed *U* test, *p* < 0.0001, *p* = 0.0005, and *p* = 0.0264 for Pirt+, CGRP+, and GFP+, respectively; Fig. 11*O*): for example, in the Pirt-Ai9 lung: 41 of the 47 (87%) NEBs comprised of more than six CGRP+ cells were innervated by tdTomato+ (i.e., Pirt+) fibers, whereas only 12 of the 25 (48%) NEBs comprised of less than or equal to six CGRP+ cells were innervated. Furthermore, 13 of the 19 (68%) NEBs without tdTomato+ innervation had less than or equal to six CGRP+ cells. We also identified five solitary CGRP+ epithelial cells in the 296 airways analyzed, only one of which was innervated by CGRP+ fibers.

## Discussion

Electrophysiological, biochemical, and transcriptomic studies of the vagal sensory innervation to the airways have identified distinct afferent subsets (Coleridge and Coleridge, 1984; Mazzone and Undem, 2016; Taylor-Clark, 2021). Selective activation or interruption of specific subsets has demonstrated their role in regulating respiratory and autonomic responses (Mazzone and Canning, 2002; Mazzone et al., 2005, 2009; Muroi et al., 2011, 2013; Tränkner et al., 2014; Chang et al., 2015; McAlexander et al., 2015; Nonomura et al., 2017; Baral et al., 2018; Chou et al., 2018). There is surprising heterogeneity in airway nociceptive reflexes (Chou et al., 2008, 2018; Muroi et al., 2013; Hooper et al., 2019), some of which can be explained by the jugular versus nodose paradigm (Taylor-Clark, 2021), but there appears to be further complexity which may depend on anatomically distinct subsets. The current gap in our knowledge of the innervation patterns of specific subsets hinders our understanding of how pathologic insults such as inflammation, infection or edema trigger reflexes that can contribute to disease morbidity.

Using Cre-mediated reporter expression to map genetically-defined sensory subsets has advantages over IHC, which may be hampered by the absence or limited expression of specific markers and poor antibody selectivity (or lack of validation). Here, cell-specific expression of GFP or tdTomato was robustly driven by the endogenous ROSA26 gene or by CAG promoters in AAV, thus providing high signal-to-noise ratios for the detection of even the thinnest fibers. Nevertheless, there are some caveats to our approach. First, tdTomato expression driven by the ROSA26 gene reflects Cre expression at any point in the neuron’s lineage, not necessarily *current* Cre expression in the adult neuron (Cavanaugh et al., 2011a; Kim et al., 2020b). Whereas AAV-mediated reporter expression only occurs if Cre is actively expressed. Second, injection/instillation with AAV vectors is unlikely to transfect all sensory neurons/fibers within that locale. For example, lung instillation of rAAV2-flex-tdTomato unexpectedly failed to label large blood vessels, although these structures likely had Cre+ fibers.

Here, we opted to image 80-μm-thick lung sections. Thus, in many cases reporter-expressing fibers were found to enter in/out of the physical plane making comprehensive structural analysis impossible. As such, it is likely that the projecting fiber length is an underestimate. Nevertheless, confirmed terminations for specific fibers were identifiable if they occurred between the upper and lower limits of the z-stacked image.

Pirt is a TRP channel regulator that is expressed in almost all sensory neurons in the vagal ganglia and DRG but not in other cell types (Patel et al., 2011; Patil et al., 2019; Kim et al., 2020b). Pirt+ fibers innervated almost all conducting airways but only ∼30% of blood vessels, indicating that afferents project to specific structures. The vagal-specific Pirt+ innervation was almost as widespread as the overall Pirt+ innervation. The conducting airways were also densely innervated by TRPV1+, 5HT_3_+, and Tac1+ fibers. TRPV1 is expressed by almost all mammalian C fibers projected from the nodose and jugular ganglia to the lungs (Ho et al., 2001; Undem et al., 2004; Hooper et al., 2016), and their activation evokes defensive reflexes (Coleridge and Coleridge, 1984; Mazzone and Undem, 2016; Taylor-Clark, 2021). In the guinea pig, TRPV1+ fibers have been identified by IHC innervating the conducting airways (Watanabe et al., 2005, 2006). In the mouse, however, TRPV1 expression is restricted to C fibers that conduct action potentials <0.75 m/s (Kollarik et al., 2003). Mouse TRPV1-expressing vagal afferents are critical for the airway hyperreactivity associated with allergic asthma and bacterial clearance in a model of pneumonia (Tränkner et al., 2014; Baral et al., 2018). Our data indicate that nociceptive vagal fibers innervate the vast majority of conducting airways, intercalating with the smooth muscle layer, and coming into close proximity with the epithelium. Although we did not co-label the vagal TRPV1+ fibers with specific nodose or jugular markers, we also observed vagal Tac1+ fibers and 5HT_3_+ fibers innervating and terminating within the same smooth muscle layer of the conducting airways as TRPV1+ fibers. Tac1, the gene that encodes preprotachykinin (precursor to the tachykinin neuropeptide substance P), is expressed in jugular TRPV1+ neurons but not nodose TRPV1+ neurons (Undem et al., 2004; Nassenstein et al., 2010; Kim et al., 2020b). Whereas 5HT_3_ is expressed exclusively in nodose neurons (Chuaychoo et al., 2005; Kim et al., 2020b). Thus, our data suggest that both nodose C fibers and jugular C fibers innervate the conducting airways through to the smallest airways. This is consistent with receptive field mapping of lung C fibers to punctate stimulation (Undem et al., 2004). The density of 5HT_3_+ fibers in the large and medium diameter airways appeared greater than for the vagal TRPV1+ or vagal Tac1+ innervation. Indeed, the density of 5HT_3_+ innervation in these airways resembled the density of Pirt+ innervation. Previously, tdTomato in the 5HT_3_-Ai9 was observed in both TRPV1+ and TRPV1-negative nodose neurons (Kim et al., 2020b), and thus much of the 5HT_3_+ and Pirt+ fiber density is likely because of the presence of TRPV1-negative fibers. The extent of the TRPV1-negative innervation was surprising given that TRPV1-negative A fibers represent only ∼10–20% of lung afferents (Coleridge and Coleridge, 1984; Mazzone and Undem, 2016), suggesting that the arborization of these afferents or C fibers conducting >0.75 m/s is extensive compared with TRPV1+ fibers. Here, we noted that multiple TRPV1+ fibers innervated the same large conducting airway, but each fiber had a relatively simple arbor.

While all studied vagal subsets innervated the conducting airways, only Pirt+, TRPV1+, and 5HT_3_+ fibers projected into the alveolar region. We therefore conclude that only nodose fibers, many (if not all) being TRPV1+, innervate these regions. Such projections only occurred from conducting airways (not blood vessels) that were <375 μm in diameter. We found no evidence that neural crest-derived sensory neurons (jugular or DRG) project into the alveolar region. These data are consistent with a recent report using vagal injections of AAV-flex-reporter vectors in TRPV1^Cre^ and Tac1^Cre^ mice (Su et al., 2021), although substance P+ fibers were occasionally observed innervating the lung parenchyma in the guinea pig (Kummer et al., 1992).

Sensory innervation of blood vessels was limited compared with conducting airways, consistent with other reports in mice (Su et al., 2021), although another study in guinea pig suggests widespread vessel innervation by substance P+ fibers (Haberberger et al., 1997). Large blood vessels were much more likely to be innervated by Pirt+ vagal fibers than smaller vessels. Although there was some 5HT_3_+ innervation of blood vessels (indicating nodose fibers), the vagal Pirt+ innervation pattern was replicated by vagal Tac1+ innervation (likely jugular fibers). Although we did not assess Tac1+ and TRPV1+ expression in the same tissue, fewer large blood vessels were innervated by vagal TRPV1+ fibers (36%) compared with vagal Tac1+ fibers (64%), suggesting the existence of a vagal Tac1+/TRPV1-negative population. In rat and guinea pig studies, capsaicin toxicity has been shown to eliminate both substance P immunoreactivity and vagal stimulation-evoked tachykinergic-mediated (i.e., neurogenic) bronchospasm and vascular leakage, suggesting that substance P is solely expressed in TRPV1+ afferents (Lundberg et al., 1983, 1984; Ellis and Undem, 1990, 1992; Baluk et al., 1992). However, not all mouse lung C fibers express TRPV1 (Kollarik et al., 2003), and others have identified Tac1+/TRPV1-negative populations within the mouse vagal ganglia (Surdenikova et al., 2012; Kupari et al., 2019; Kim et al., 2020b). The identity and function of these putative vagal Tac1+/TRPV1-negative fibers is unclear.

Using latex beads, we identified the pulmonary arteries and pulmonary veins in Pirt-Ai9 mice. Pirt+ fiber innervation was more common for both larger arteries and larger veins. Indeed Pirt+ fibers innervated almost all medium and large diameter veins but none of the smaller veins. Although we did not perform the latex labeling in the other strains, it is likely that much of the sensory innervation of large veins is Tac1+ (but not necessarily TRPV1+).

Importantly, sectioning or ablation of vagal afferents does not eliminate afferent fibers within the lung (Lundberg et al., 1983; Baluk et al., 1992). Conventional retrograde tracing (e.g., DiI, fast blue) in guinea pig (Kummer et al., 1992; Oh et al., 2006), rat (Groth et al., 2006), and mouse (Dinh et al., 2004; Nassenstein et al., 2010) indicate that DRG afferents also project to the lungs, as do a few neurons within the esophageal myenteric plexus (Fischer et al., 1998). Using AAV injected into the DRG, we demonstrate direct evidence that DRG Pirt+ fibers innervate conducting airways of all sizes, but such innervation is much more common in larger airways. DRG Pirt+ fibers also innervated blood vessels, but this was largely restricted to those >376 μm in diameter. The overwhelming predominance of vagal versus DRG innervation observed here is consistent with retrograde tracing in multiple species (Kummer et al., 1992; Dinh et al., 2004; Nassenstein et al., 2010; McGovern et al., 2012). Nonetheless, the density of DRG innervation of large airways was particularly striking. A total of 70% of large airways and 10% of medium airways were also innervated by DRG Tac1+ fibers, but the DRG TRPV1+ innervation was very sparse. Similarly, more blood vessels were innervated by DRG Tac1+ fibers compared with DRG TRPV1+ fibers. Unfortunately, there is a lack of consensus regarding protein expression in DRG lung afferents. TRPV1 appears widespread in guinea pig and rat DRG lung afferents (Groth et al., 2006; Oh et al., 2006), but TRPV1 expression has been reported in ∼10% of mouse DRG lung afferents by IHC (Dinh et al., 2004) and ∼34% by single neuron RT-PCR (Nassenstein et al., 2010). Our data suggest there is a population of DRG Tac1+/TRPV1-negative fibers innervating the mouse lung, and this is consistent with the innervation of other visceral organs (Surdenikova et al., 2012; Hockley et al., 2019). The contribution of fibers projected from esophageal myenteric plexus neurons (Fischer et al., 1998) to the fibers labeled in the Pirt-Ai9 or Tac1-Ai9 lung is currently unknown.

Interestingly, DRG innervation of blood vessels by Pirt+ or Tac1+ fibers was more likely than vagal innervation to penetrate through the muscle layers to come into close proximity to the endothelium. Instead, vagal Pirt+ or vagal Tac1+ fibers seemed to innervate the outer muscle layers. The vascular smooth muscle layer of some intrapulmonary veins is surrounded by a discontinuous coat of cardiomyocytes (Mueller-Hoecker et al., 2008). It is currently unresolved whether such cardiomyocytes receive differential sensory innervation compared with the vascular smooth muscle.

Little is known of the physiological role of DRG afferents innervating the mouse lungs. Activation of TRPV1+ DRG afferents in guinea pigs and rats evokes sympathetic reflexes to the lung and heart (Oh et al., 2006; Shanks et al., 2018). Inhibition of capsaicin-evoked sympathetic reflexes then reveals a minor DRG-mediated neurogenic bronchospasm (Saria et al., 1985). As mentioned above, most DRG lung afferents do not express TRPV1 in the mouse, thus it is not clear whether they too would evoke similar reflexes.

CGRP is another sensory neuropeptide that is preferentially expressed in jugular neurons compared with nodose neurons (Springall et al., 1987; Helke and Hill, 1988; Zhuo et al., 1997). Similar to Tac1, CGRP+ fibers innervated almost all conducting airways and some blood vessels. However, unlike Tac1+ fibers, some CGRP+ fibers projected into the alveolar region. All of these CGRP+ fibers also expressed TRPV1. We should note, however, that the majority of TRPV1+ fibers that projected to the alveolar regions lacked CGRP. Given that only nodose fibers projected into the alveolar region, we conclude that some of these nodose TRPV1+ fibers express CGRP but not substance P, consistent with reports that CGRP does not match Tac1 expression in mice (Wang et al., 2017; Kupari et al., 2019). However, this conclusion conflicts with tracing studies that suggest ∼1% of airway-specific nodose neurons express CGRP in the guinea pig and rat (Springall et al., 1987; Kummer et al., 1992; Undem et al., 2004).

CGRP is also a marker of specialized epithelial cells, whose clusters are termed NEBs. NEBs occur in multiple mammalian species and are innervated by multiple sensory nerve subtypes (Brouns et al., 2003, 2009; Adriaensen et al., 2015). NEBs are thought to act as part of the sensory system, although their precise role has been debated for decades (Adriaensen et al., 2015; Mazzone and Undem, 2016; Sui et al., 2018). Overall, we found ∼74% of NEBs were innervated, similar to previous studies in mice (Chang et al., 2015) and rats (Larson et al., 2003). Consistent with previous reports, we found NEBs were innervated by at least two types of (Pirt+) sensory fibers: CGRP+ and CGRP-negative fibers (Brouns et al., 2003, 2009). These CGRP-negative NEB-innervating afferents are likely myelinated fibers that express the markers P2ry1 (Chang et al., 2015) or Calbindin 1 (Su et al., 2021). Because of their reported (Brouns et al., 2003, 2009) lack of expression of the nodose marker P2X_2_ (Kim et al., 2020a), CGRP+ fibers innervating NEBs were originally thought to be projected from DRG neurons, but our data suggest that many are TRPV1+ fibers projected from the vagal ganglia. As such, we suggest many CGRP+ fibers innervating NEBs are from jugular neurons.

Lung instillation of rAAV demonstrated that both Pirt+ and TRPV1+ airway afferents have central projections to the nTS and the area postrema. Importantly, no airway afferents projected to the Pa5. This is consistent with other reports using rAAV lung instillation in mice (Kim et al., 2020b; Su et al., 2021), despite observations that vagal injection of AAV9 causes reporter expression in jugular afferents projecting to the nTS and the Pa5 (Kim et al., 2020b; Su et al., 2021). Furthermore, airway instillation of herpes simplex viral vectors in guinea pigs and rats have traced airway jugular (but not nodose) afferent projections to the Pa5 (McGovern et al., 2012, 2015; Driessen et al., 2015) and functional imaging has demonstrated evidence of this pathway in humans (Farrell et al., 2020). It is possible that only jugular afferents innervating organs other than the lungs project to the Pa5 in mice. Nevertheless, it has been postulated that the AAV instillation in mice fails to transfect the trachea and large conducting airways where the majority of jugular afferents are presumed to be primarily located (Su et al., 2021). But here we provide evidence that Tac1+ afferents (likely jugular) are found in great abundance throughout the conducting airways, and it is probable that even a low number of reporter+ terminals would be detectable in the Pa5 using confocal microscopy. Alternatively, it is possible that the lack of airway-Pa5 circuitry in the mouse is because of AAV tropism. Details are presently lacking for the airway-Pa5 circuitry, but evidence suggests that tropism is a factor for AAV-mediated labeling of DRG lung afferent innervation. We have found that DRG injection of Pirt^Cre^ with AAV9-flex-tdTomato revealed a substantial tdTomato+ DRG innervation of large conducting airways. Nevertheless, lung instillation of Pirt^Cre^ with rAAV2-flex-tdTomato (which induced robust tdTomato expression in vagal neurons innervating the large airways) traced no fibers back to the DRG. This is consistent with other rAAV2 studies in the mouse lung that failed to trace to DRG neurons (Su et al., 2021), despite the fact that conventional retrograde tracers such as fast blue or DiI identify many lung-labeled DRG afferents (Kummer et al., 1992; Dinh et al., 2004; Oh et al., 2006; Nassenstein et al., 2010). AAV tropism for afferent subsets may be dependent not only on the intrinsic properties of the vector and the neuron (Mason et al., 2010; Jacques et al., 2012; Kudo et al., 2021) but also perhaps on the properties of the afferent terminal within the specific lung tissue. Given the widespread use of AAVs, more work is needed to explore afferent subset-specific tropism *in situ*.

**Figure 12.**
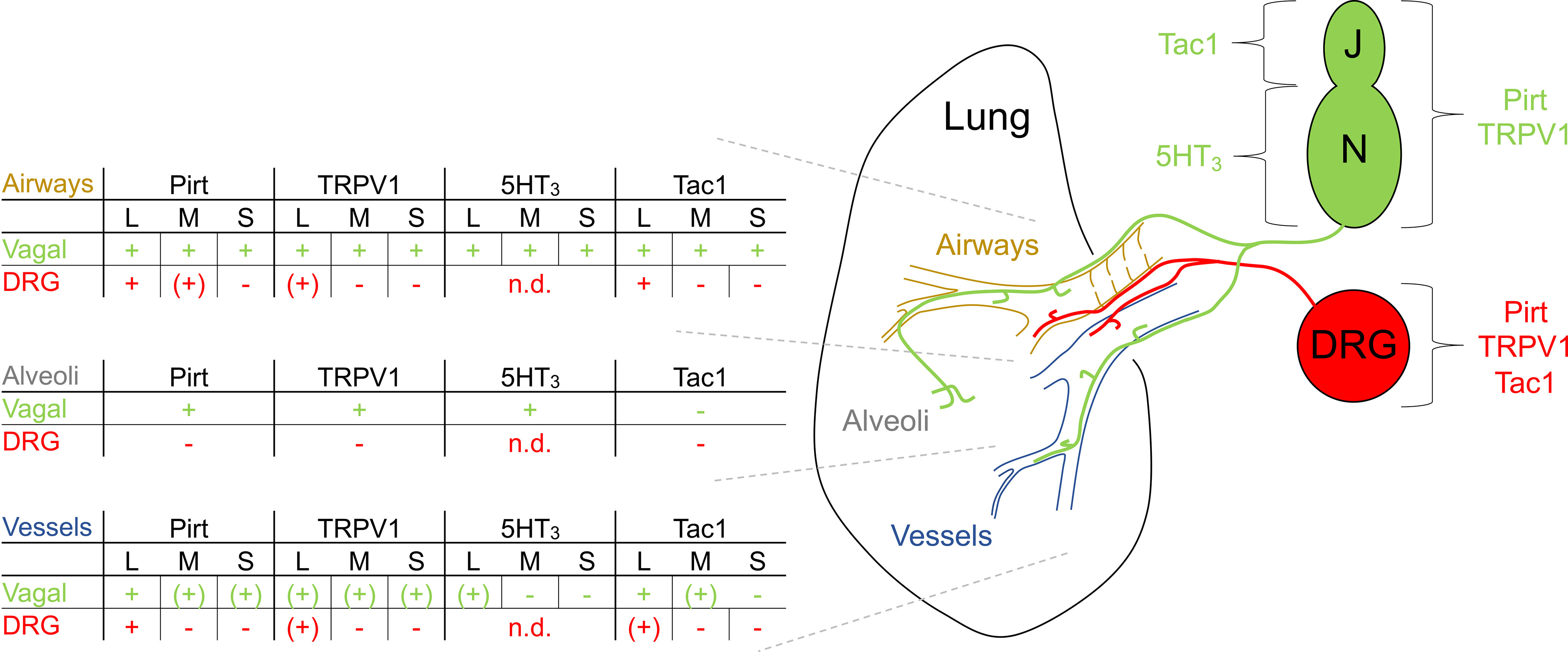
Overview of the vagal and DRG sensory innervation of the mouse lung. Schematic identifies three lung structures [conducting airways (brown), blood vessels (blue), and alveoli (gray)] and their innervation by vagal Pirt+, TRPV1+, Tac1+ (predominantly jugular only) and 5HT3+ (nodose only) fibers (green) and DRG Pirt+, TRPV1+, Tac1+ fibers (red). Airways and blood vessels are subcategorized into large (L, >376 μm in diameter), medium (M, 176–375 μm in diameter) and small (S, 0–175 μm in diameter) groups. Innervation is semi-quantified for specific structures: + denotes common occurrence, (+) denotes rare occurrence, - denotes absence, and n.d. denotes not determined.

In summary, our data indicate that sensory innervation of structures within the lung is complex (Fig. 12). We have identified substantial differences in the innervation patterns of nodose and jugular afferents, particularly for fibers projecting into the alveolar region. We have also identified specific innervation patterns for the arterial and venous vessels in the pulmonary circulation. Lastly, we have identified a robust DRG innervation of large conducting airways and blood vessels, which partially expresses Tac1, but lacks TRPV1 expression. As discussed above, the afferent innervation of the mouse lung is somewhat different to that of other mammals such as the guinea pig – in particular the contribution by TRPV1-negative C fibers. Mice also lack a major nodose Aδ fiber subset that innervates the trachea (Hennel et al., 2018; Kim et al., 2020a), and mice lack a cough reflex to canonical tussive stimuli (Plevkova et al., 2021) and have limited neurogenic inflammatory responses (Baluk et al., 1999). Nevertheless, the genetic tools available for the mouse provide anatomically relevant information regarding distinct nociceptive and non-nociceptive subsets whose activation evokes specific respiratory and cardiovascular reflexes (Coleridge and Coleridge, 1984; Mazzone and Undem, 2016; Taylor-Clark, 2021).

Here, we have focused on the nodose-jugular paradigm for identifying distinct subsets, but there remains the possibility that other gene expression patterns may govern anatomically distinct subsets currently grouped together, e.g., nodose TRPV1+ fibers innervate the conducting airways, the alveolar region and some blood vessels – are these the same functional fiber type or three distinct subtypes? After all, exogenous and endogenous stimuli (including physical stretch, pollutants, irritants, inflammatory mediators, autacoids, and edema) would be expected to differ greatly across these regions. In addition, the expression of many sensory nerve markers, including Tac1 and TRPV1, and the lung fiber innervation patterns themselves are plastic and can be modulated by inflammation and infection (Hoyle et al., 1998; Hunter et al., 2000; Carr and Undem, 2001; Zhang et al., 2008; Lieu et al., 2012). More work is needed to determine the role of specific vagal and DRG subsets innervating distinct anatomic sites in evoking reflex responses in health and disease.

## Synthesis

Reviewing Editor: Christopher Colwell, UCLA School of Medicine Decisions are customarily a result of the Reviewing Editor and the peer reviewers coming together and discussing their recommendations until a consensus is reached. When revisions are invited, a fact-based synthesis statement explaining their decision and outlining what is needed to prepare a revision will be listed below. The following reviewer(s) agreed to reveal their identity: Brendan Canning, Wolfgang Kummer.

We all agree that the authors have employed several innovative approaches to identify projections of presumed afferent nerve subtypes to various structures in the airways and lungs. Stunning images were produced and several interesting inferences drawn.

Nevertheless, the reviewers raised important concerns that will need to be addressed.

Reviewer 1:

Specific comments

1) The images produced were beautiful. There are so many images and so many different transgenic approaches and labeling approaches used that it is often difficult to remember what the point is of the many figures. I don't have a good sense of how to construct it, but a summary figure would be helpful.

2) I appreciate the authors noting the various limitations to their approaches, including neurons that are not labeled and the trade off associated with using wholemounts (and thus the lack of detailed structural assessments of terminals). This may explain why the data seem to contradict or at least fail to reproduce the results of many previous studies. A few examples are presented below.

3) Studies done in rats and guinea pigs suggest that nearly all neurokinin-containing afferents (both vagal and spinal) innervating the airways and lungs are capsaicin-sensitive: Lundberg et al., Acta Physiologica Scand., 1983 Nov;119(3):243-52; Lundberg et al., Cell Tissue Res. 1984;235(2):251-61).

4) Neurokinin-containing spinal afferents project to the airways and lungs of rats and guinea pigs: Saria et al., Acta Physiol Scand. 1985 Nov;125(3):407-14; Kummer et al., Neuroscience. 1992 Aug;49(3):715-37.

5)Nearly all neurokinin-containing sensory nerve terminals in the trachea of rats is vagal in origin based on nerve cuts and immunohistochemistry: McDonald et al.; Neurocytol. 1988 Oct;17(5):605-28; Baluk et al., J Comp Neurol. 1992 May 22;319(4):586-98.

6) Even in mice, to the extent there is neurogenic plasma leakage (sensitive to NK1 receptor antagonists), it would seem to depend upon capsaicin-sensitive nerves: Baluk et al., Br J Pharmacol. 1999 Jan;126(2):522-8.

7) TRPV1+ spinal afferents do seem to project to the most distal airways (Groth et al., Respir Res. 2006 Jul 1;7(1):96) and also innervate the smallest arterioles (Haberberger et al., J Appl Physiol. 1997 Feb;82(2):426-34).

8) Could some of the TAC1+/ TRPV1- innervation of the airways in mice project from the myenteric plexus of the esophagus? Capsaicin only partially depletes the esophagus of SP-IR (Lundberg et al., Acta Physiol Scand. 1983 Nov;119(3):243-52). Esophageal myenteric plexus neurons can express SP (Sternini et al., Gastroenterology. 1989 Aug;97(2):348-56). Myenteric plexus neurons have been shown to project to the adjacent airways (Fischer et al., J Comp Neurol. 1998 May 11;394(3):326-34).

9) To the extent the results summarized in the MS fail to reproduce the results published previously, some of this discrepency may be attributable to species differences. It may be useful to highlight the many peculiarities of murine (and rat) lung innervation, for example: seemingly No Adelta cough receptors (Mazzone et al., J Neurosci. 2009 Oct 28;29(43):13662-71); No cough response to capsaicin challenges in conscious mice (Zhang et al., Am J Physiol Regul Integr Comp Physiol. 2017 May 1;312(5):R718-R726).

Reviewer 2:

I have several major concern with the manuscript in its present version.

A. Methodological

1. Of utmost importance; Numbers of animals and experiments are missing from the methods section and from the figures legends when quantitative data are shown (mostly percentages). Partly, such information is included in the main text of the results section [e.g. line 329f: PirtCre mice (n=3 animals, n=7 slices)], but this does not clarify how many of them were evaluated for each parameter shown in the different figure panels. In addition, there are vague statements at other places (line 401: For some Tac1-Ai mice). Throughout, “N” (animals, sections being evaluated, number of objects - e.g. airways - being evaluated) must be accurately specified. This should also include information how many animals of a given strain were subjected to further methods, e.g. immunolabeling.

2. Characterization of models. The genetic models look elegant and impressive, but as we all know, not each (carefully said) genetic model results in a line that matches our expectation. A thorough characterization of each model is warranted - if that had been given before, an appropriate reference suffices.

3. Controls. The authors used immunolabeling; I have not found a description of methodological controls of this approach.

4. Statistics. The authors used student’s t-test throughout (line 266; line 989) without providing evidence of normal distribution of data. Rather, the violin plots depicted in Fig. 2M,N evidently shown a non-normal data distribution, and these data were subjected to t-test. The analyses need to be re-done with non-parametric tests, and precise p-vales rather than “ns” or “*” shall be provided (and, of course, “N"; see above).

5. Method details often remain vague and need to be specified. Examples: Line 113: “Specific alleles were confirmed by genotyping per developers’ instructions.” Full details must be made available. Either provide a literature reference, if it was really conducted exactly (!) according to this published protocol, or provide the full protocol here. There are further, less prominent examples, e.g. line 133f “equal volumes”: how much?, and so on. Please work carefully throughput the entire methods description to make sure that description allows others, in principal, to repeat these experiments.

B. Results

6. It appears as if the authors have overlooked the fact that pulmonary veins in mice (like in other rodents) have an outer sheath of cardiac muscle extending deep into the lung. At these places, a single layer of smooth muscle is located between this outer cardiomyocytes and the endothelium. These veins are innervated, but they are never mentioned in the present manuscript. Fig. 3L,M, 4O and others depict such veins (even the DAPI stain allows to identify the cardiomyocyte nuclei), but the authors speculate about nerve fibers penetrating multiple layers of smooth muscle (rather than the cardiomyocyte sleeve). I recommend to go back to the original material, identify the cardiomyocytes, and then describe innervation of these compartments.

7. Neuroendocrine cells of the airway epithelium occur clustered (= neuroepithelial body = NEB) or as solitary cells, and there appear to be differences between NEB and solitary cells. The present authors, however, speak about “NEB” even if only a single cell builds up a “body” (line 498). Please provide information separately for solitary cells and NEB; as the raw data are already collected, this does not require further experimentation

C. Writing and intellectual content

8. A line of airway innervation research utilizing elegant genetic models (reporter expression, optogenetics, TRPV1-based cell ablation etc.) is fully neglected here, despite its high relevance to the present study. This also includes a study (PMID: 28002412) addressing the difference between nodose and jugular vagal neurons, very similar to the present study. It is surprising that these prominently published papers - Cell (PMID: 25892222), Nature (PMID: 28002412), Nat Med (PMID: 29505031), PNAS (PMID: 25049382) - are not mentioned here. Instead, the manuscript reads as if this were the first study of airway innervation utilizing such genetic models. These reports need to be adequately addressed in the present manuscript.

9. Lines 91 to 97 (end of introduction) represent a summary of findings rather than introduction.

10. The first paragraph of the discussion (lines 519-537) has rather the character of an introduction rather than discussion. In view of the overall length of the discussion, it can be omitted.

11. Three times, the authors use “data not shown” for discussing their findings (lines 556, 704, 710). This should be avoided: If these data are relevant, shown them (or don’t draw conclusions out of them).

Minor

12. The term “alveolar space” is generally used for the alveolar lumen filled with air and lined with surfactant. It is not innervated. Yet, phrases such as “projected out into the alveolar space” (line 357) are commonly used in the text. It shall be replaced by “alveolar region", “alveolar septa” or simply “alveoli", or something similar.

13. Table 1: Secondary antibodies are not directed against animals (e.g. anti-rabbit), but against immunoglobulins (e.g. anti-rabbit Ig, and so on).

## Author Response

Authors' introduction: We would like to thank the editorial office and reviewers for their constructive critique of our manuscript. We have addressed each point in detail below and have accordingly made numerous changes to the manuscript, particularly to remain within the word limit of the introduction and discussion.

Significance Statement Comments for Author (Required):

The authors studied sensory lung innervation in nice using an impressive number of genetic models and elegant microscopic imaging. Despite there are already not just few other studies addressing sensory lung innervation, the present report adds several new aspects and promotes our understanding of the subject.

Comments on the Visual Abstract for Author (Required):

The ms would benefit from a visual abstract.

Authors’ response: We have provided a summary image (Fig 12)

Synthesis Statement for Author (Required):

We all agree that the authors have employed several innovative approaches to identify projections of presumed afferent nerve subtypes to various structures in the airways and lungs. Stunning images were produced and several interesting inferences drawn.

Nevertheless, the reviewers raised important concerns that will need to be addressed.

Reviewer 1:

Specific comments

1) The images produced were beautiful. There are so many images and so many different transgenic approaches and labeling approaches used that it is often difficult to remember what the point is of the many figures. I don’t have a good sense of how to construct it, but a summary figure would be helpful.

Authors’ response: We have now included a summary figure.

2) I appreciate the authors noting the various limitations to their approaches, including neurons that are not labeled and the trade off associated with using wholemounts (and thus the lack of detailed structural assessments of terminals). This may explain why the data seem to contradict or at least fail to reproduce the results of many previous studies. A few examples are presented below.

Authors’ response: We thank the reviewer for highlighting these studies, and we will address each study in detail below. Contrary to the reviewer’s statement, we respectfully suggest that our data are not inconsistent with many of the listed studies, especially as many focus on the trachea (not the lung) and non-murine species, both of which are likely determining factors.

First, though, we should note that there are caveats associated with these highlighted studies, many of which relied on substance P antibodies which had not all been validated; and only a few used objective and unbiased methods to quantify the substance P+ fibers innervating airway structures. Instead, most presented images as representative of the total population. How representative these actual images are is sometimes unclear.

3) Studies done in rats and guinea pigs suggest that nearly all neurokinin-containing afferents (both vagal and spinal) innervating the airways and lungs are capsaicin-sensitive:

Authors’ response: The Lundberg et al (1983) paper presents evidence, in guinea pigs, that 90% of substance P expression in the trachea and lungs is eliminated by capsaicin toxicity, indicating that most (but perhaps not all) substance P is expressed in TRPV1+ afferents. Interestingly only 60% loss occurs with vagal injection of capsaicin, suggesting (but not proving) some substance P+ fibers may be projected from DRG neurons (or perhaps from neurons within the myenteric plexus - see below). IV injection of capsaicin evoked complex changes in airway pressure, some of which was likely neurogenic in origin (atropine-insensitive). This was eliminated by systemic capsaicin toxicity, drawing the circular conclusion that capsaicin evokes neurogenic bronchospasm via capsaicin toxicity-sensitive nerves.

Lundberg et al. (1984) present evidence in guinea pig, rat and cat of substance P+ immunoreactivity in fibers innervating the bronchi through to the terminal bronchioles. There was a “marked decrease” in substance P+ fibers in the lungs after capsaicin toxicity, but there were definitely residual substance P+ fibers.

In our study, we did not use intersectional genetics to identify overlap of Tac1 and TRPV1 expression. A side-by-side comparison of the expression patterns generated by the Tac1-AAV-GFPvagal and TRPV1-AAV-GFPvagal approaches suggest that both vagal Tac1+ and vagal TRPV1+ fibers innervate conducting airways (down to the smallest diameter, i.e. terminal bronchioles) and blood vessels. Clearly, we also observed vagal TRPV1+ but not vagal Tac1+ fibers innervating the alveolar regions, indicating vagal TRPV1+/Tac1- fibers. But there is little evidence in our data suggesting there is a substantial vagal TRPV1-/Tac1+ population innervation of the conducting airways. Our data does suggest that there are possibly some vagal TRPV1-/Tac1+ fibers innervating the large blood vessels (Fig 1H), but without simultaneously assessing TRPV1 and Tac1, it is difficult to be definitive. On the other hand, there does seem to be a TRPV1-/Tac1+ population projected from DRG to the large airways (∼70% of these structures, Fig 1E). However, it is important to consider that there are relatively fewer large conducting airways compared to medium and small structures (Fig 1C). Thus at the whole lung level, the putative TRPV1-/Tac1+ DRG population is a relatively minor one compared to (for example) the vagal TRPV1+ innervation. Indeed, the 10% residual substance P+ described by Lundberg et al (1983) could represent this minor TRPV1-/Tac1+ DRG population.

Furthermore, we had emphasized in the discussion that unlike guinea pig (Undem et al, 2004, PMID 14978204) and rats (Ho et al, 2001, PMID 11504584; Hooper et al, 2016, PMID 26718787), not all vagal C-fibers innervating the mouse lung are TRPV1+ (Kollarik et al, 2003, PMID 12909686). It is not known if vagal TRPV1- C-fibers innervating the mouse lung express Tac1. But we note that TRPV1-/Tac1+ sensory neurons in general are more common in mice than other mammals (e.g. Surdenikova et al, 2012, PMID 22937918; Cavanaugh et al, 2011, PMID 21451044; Kim et al, 2020, PMID 32060036). Thus if there really is a vagal TRPV1-/Tac1+ population innervating the mouse airways, it would be consistent with previous mouse data, which as noted above is different from guinea pig and rat described by Lundberg et al (1983, 1984). This concept has now been highlighted in the text.

We did use a counterstain for CGRP in the TRPV1-AAV-GFPvagal studies, indicating a small population of vagal TRPV1-/CGRP+ fibers innervating the conducting airways and blood vessels (although not the alveolar region). Nevertheless, it is important to not extrapolate Tac1 expression from CGRP expression in the mouse (Kupari et al, 2019 PMID 31116992; Mazzone et al, 2020, PMID 31630330).

4) Neurokinin-containing spinal afferents project to the airways and lungs of rats and guinea pigs:

Authors’ response: The Saria et al. (1985) paper investigated the guinea pig lung and found a 35% reduction in total substance P content after stellatectomy, suggesting a contribution by DRG afferents. They also presented images of substance P+ fibers innervating a medium-diameter conducting airway, and then stated that surprisingly the number of substance P+ fibers did not change after stellatectomy. This inconsistency may be a function of their approach: small tissue specimens were studied for the substance P IHC with no statistical approaches or n numbers mentioned. Importantly, capsaicin-evoked bronchospasm was reduced 56% by stellatectomy, suggesting a role of DRG afferents. Furthermore, electrical stimulation of the stellate in the presence of 6-OHDA, phentolamine and propranolol (to block sympathetic reflexes), evoked bronchospasm that was atropine-independent. This bronchospasm was completely abolished by capsaicin-pretreatment, suggesting that TRPV1+ afferents are completely responsible for the DRG-mediated neurogenic bronchospasm. To the extent that this paper shows there is DRG innervation of the guinea pig lung by DRG afferents (and that some of it expresses substance P), we would argue that this is consistent with our current data. Saria et al do, however, suggest that the DRG substance P expression occurs exclusively in capsaicin-sensitive fibers, which is not the case in our studies (our data indicates a small population of DRG TRPV1-/Tac1+ fibers). Previous studies have shown that TRPV1 expression in DRG lung afferents is much lower in the mouse compared to guinea pig: Oh et al (2006 PMID 16581869) demonstrated that 61% of guinea pig DRG lung afferents expressed TRPV1, whereas TRPV1 expression in mouse DRG innervating the lung was ∼10% reported by Dinh et al (2004 PMID 15522699) using immunohistochemistry, and ∼34% (single cell RT-PCR) reported by Nassenstein et al (2010 PMID 20937710). We have expanded on this species difference in the discussion

We do note, however, that Dinh et al reported that all mouse lung-labeled substance P+ DRG afferents also contained TRPV1 - and this is inconsistent with our data that the Tac1+ DRG innervation appears more widespread than TRPV1+ innervation. This discrepancy is not due to the labeling of fibers that only express Tac1 during development, because the Tac1+ DRG fibers were detected using AAV-mediated expression (which relies on current cre expression). Overall, the immunohistochemistry presented by Dinh et al indicate that only ∼7% of DRG lung afferents expressed substance P, and perhaps this relatively low number is due to conservative assessment of substance P+ vs. substance P- neurons. Looking at their fig 1c, there is not a massive difference in the fluorescence of a substance P+ vs. substance P- neuron - thus potentially contributing to false negative assessments. The AAV-cre/lox system used in the current study produces a much more definitive signal to noise ratio. We have expanded on this in the discussion.

Kummer et al. (1992) performed an extensive analysis of guinea pig innervation of the trachea and lung using retrograde tracers, demonstrating that many DRG and jugular afferents traced from the airways express substance P, while few nodose airway afferents express substance P. The authors also stained airway tissue for substance P and found many substance P fibers (although their origin was not determined) innervating the epithelial layer of trachea, bronchi and bronchiole (and some blood vessels too). These data are all consistent with our data, and we had referenced this important study in the discussion. We should note that the authors also presented a single image of a substance P+ fiber innervating the alveolar region, and the text stated that “a few SP/CGRP-IR and SP/NKA-IR axons ran irregularly through the peripheral lung parenchyma”. As mentioned above, we found some CGRP+ fibers but no Tac1+ fibers innervating the alveolar region. It is difficult to assess in the Kummer et al study the frequency of the substance P+ fibers vs. the CGRP+ fibers innervating this region in the guinea pig, and so we are apprehensive to over litigate this issue, but it is possible differences between guinea pig and mice may underlie any possible differences in this regard. We have addressed this is the discussion.

5) Nearly all neurokinin-containing sensory nerve terminals in the trachea of rats is vagal in origin based on nerve cuts and immunohistochemistry:

Authors’ response: McDonald et al. (1988) investigated vagal and SLN electrical stimulation in the rat, and found that this evoked extravasation in the trachea and bronchi, which was dependent on jugular neurons. This is consistent with a peptidergic role of jugular but not nodose afferents in neurogenic inflammation. They also found substance P fibers in the trachea (innervating epithelium, arterioles, not many venules), but the authors did not determine their origin. This paper does not present any evidence (positive or negative) regarding the existence or non-existence of DRG sensory fibers innervating the airways or contributing to neurogenic inflammation. Furthermore, their data is solely focused on the trachea, whereas our data is solely focus on the intrapulmonary airways.

The Baluk et al. (1992) paper presents beautiful evidence of substance P+ fibers innervating the epithelium, arterioles and occasionally venules in the rat trachea. Capsaicin-pretreatment reduced substance P+ fibers by 96%, and capsaicin-evoked extravasation in the trachea was abolished by capsaicin-pretreatment. In addition, unilateral vagotomy (either ipsilateral or contralateral) reduced substance P+ fibers in the trachea by 50%, suggesting all the substance P+ fibers in the trachea were vagal in origin. This rat data in the trachea is not a direct comparison with our mouse lung data. Nonetheless, Baluk et al also showed that ipsilateral vagotomy reduced bronchial substance P+ fibers by 50% but contralateral vagotomy had no effect, suggesting the possibility that substance P+ fibers from non-vagal sources contributed to bronchial substance P+ innervation. This is consistent with our data that DRG Tac1+ fibers innervate the large intrapulmonary airways. We have addressed this is the discussion.

6) Even in mice, to the extent there is neurogenic plasma leakage (sensitive to NK1 receptor antagonists), it would seem to depend upon capsaicin-sensitive nerves:

Authors’ response: Baluk et al. (1999) investigated neurogenic plasma leakage in the mouse, and showed that neither IV capsaicin nor IV substance P evoked extravasation in the trachea or lung under resting conditions. After inhibition of ACE and NEP, IV capsaicin did induce extravasation in the trachea but, again, not in the lung. We respectfully disagree with the reviewer that this study determined that only TRPV1+ nerves mediate what little neurogenic inflammation does occur, because the study focuses on capsaicin-evoked neurogenic inflammation rather than all stimuli of neurogenic inflammation (e.g. evoked by electrical stimulation of the vagus and/or the stellate). We should note that this issue was more definitively addressed in the guinea pig by Ellis & Undem (1990 PMID 2085710, 1992 PMID 1323657), who showed that vagal stimulation caused atropine-insensitive neurokinin-dependent bronchospasm that was 100% eliminated by capsaicin pretreatment, indicating that all vagal substance P+ fibers expressed TRPV1. However, as mentioned above, all guinea pig airway C-fibers express TRPV1 (Ricco et al, 1996, PMID 8910234; Undem et al, 2004, PMID 14978204), whereas only some mouse lung C-fibers express TRPV1 (Kollarik et al, 2003, PMID 12909686). Thus it is difficult to extrapolate from the guinea pig to the mouse in terms of the role of TRPV1+ fibers mediating all neurogenic inflammation. We have addressed this is the discussion.

7) TRPV1+ spinal afferents do seem to project to the most distal airways (Groth et al., Respir Res. 2006 Jul 1;7(1):96) and also innervate the smallest arterioles (Haberberger et al., J Appl Physiol. 1997 Feb;82(2):426-34).

Authors’ response: The Groth et al. study used retrograde tracers applied to the costal pleura or instilled into the airways to label DRG and vagal neurons in the rat. Both approaches labeled DRG neurons (∼30-40% were TRPV1+). This % of rat DRG lung afferents expressing TRPV1 appears greater than our quantification of the lung structures innervated by DRG TRPV1+ fibers in the mouse. The relatively high % of TRPV1 expression in lung DRG in the Groth et al rat data is similar to the guinea pig data by Oh et al (2006 PMID 16581869) mentioned above. But as we stated in the discussion there is a lack of consensus regarding TRPV1+ expression in DRG airway afferents in the mouse. Our data is more in line with Dinh et al (2004 PMID 15522699) which found ∼10% of DRG lung afferents expressed TRPV1 using immunohistochemistry, compared to the ∼34% (single cell RT-PCR) reported by Nassenstein et al (2010 PMID 20937710). These references are included in the discussion.

We would disagree, however, that the Groth et al study definitely established that the DRG afferents projected to the distal airways, because it is impossible to know the final innervation of the traced fiber: the lung instillation could have labeled only TRPV1+ DRG in the large airways, and the costal pleural application could have labeled only TRPV1+ DRG afferents that innervated the parietal pleura rather than the visceral pleura - i.e. the retrograde tracer labeled afferents from multiple anatomical sources which cannot be separate within the immunohistochemistry analysis. In contrast, our work directly determines the expression of markers within the actual anatomical location in the lung.

Our data investigating lung DRG afferents in general (using the Pirt-AAV-tdTDRG approach) suggest that DRG afferents innervate ∼10% of small conducting airways. Thus, while this number is much lower than the ∼80% of small conducting airways innervated by vagal pirt+ afferents, it does indicate evidence of DRG afferents projecting into the distal lung. That these nerves in our study lack TRPV1 may due to methodological differences in detection methods: cre-based reporter vs. immunohistochemistry (which for TRPV1 antibodies are somewhat unreliable). We have now clarified this in the discussion.

Haberberger et al. (1997) performed substance P immunohistochemistry on guinea pig lung and found substance P+ fibers innervating all pulmonary arteries and arterioles, and some venules (mostly larger venules). There is no evidence presented in this paper that the substance P fibers innervating the pulmonary vasculature are projected from the DRG - i.e. no sectioning/ablation. Thus we respectfully disagree with the reviewer that Haberberger et al show evidence of DRG substance P+ innervation of the smallest arterioles. Also, this data investigates guinea pig lung, not mouse, and we have addressed the likelihood of species differences in earlier sections.

8) Could some of the TAC1+/ TRPV1- innervation of the airways in mice project from the myenteric plexus of the esophagus? Capsaicin only partially depletes the esophagus of SP-IR (Lundberg et al., Acta Physiol Scand. 1983 Nov;119(3):243-52). Esophageal myenteric plexus neurons can express SP (Sternini et al., Gastroenterology. 1989 Aug;97(2):348-56). Myenteric plexus neurons have been shown to project to the adjacent airways (Fischer et al., J Comp Neurol. 1998 May 11;394(3):326-34).

The Tac1-Ai9 mouse expressed tdTomato in all cells that express (or have ever expressed) Tac1, thus it is possible that some of the tdTomato-expressing fibers are derived from Tac1-expressing neurons in the esophageal myenteric plexus. As we mentioned above, we did not label Tac1 and TRPV1 in the same lungs, so we cannot confirm the existence of TRPV1-/Tac1+ in the Tac1-Ai9 mice. Based upon the similarities of the TRPV1-AAV-GFPVagal data and the Tac1-AAV-GFPVagal, we have no evidence of a substantial TRPV1-/Tac1+ fiber population in the vagal lung innervation. We do, however, think that there might be a small DRG TRPV1-/Tac1+ fiber population innervating the medium and large conducting airways, but these were identified by direct DRG injection of AAV-flex-tdTomato and thus would not label myenteric plexus neurons. We acknowledge the possibility that the Tac1-Ai9 could label fibers derived from the myenteric plexus neurons and have included this in the text.

9) To the extent the results summarized in the MS fail to reproduce the results published previously, some of this discrepency may be attributable to species differences. It may be useful to highlight the many peculiarities of murine (and rat) lung innervation, for example: seemingly No Adelta cough receptors (Mazzone et al., J Neurosci. 2009 Oct 28;29(43):13662-71); No cough response to capsaicin challenges in conscious mice (Zhang et al., Am J Physiol Regul Integr Comp Physiol. 2017 May 1;312(5):R718-R726).

Authors’ response: We agree with the reviewer that the noted difference in the existence of TRPV1-/Tac1+ in the DRG innervation and possibly in the vagal innervation of blood vessels is likely due to species differences between the mouse used here and the guinea pig most often investigated in the literature. We also agree that the mouse has other differences in the innervation and evoked reflex responses which we have now highlighted in the penultimate paragraph of the discussion.

Reviewer 2:

I have several major concern with the manuscript in its present version.

A. Methodological

1. Of utmost importance; Numbers of animals and experiments are missing from the methods section and from the figures legends when quantitative data are shown (mostly percentages). Partly, such information is included in the main text of the results section [e.g. line 329f: PirtCre mice (n=3 animals, n=7 slices)], but this does not clarify how many of them were evaluated for each parameter shown in the different figure panels. In addition, there are vague statements at other places (line 401: For some Tac1-Ai mice). Throughout, “N” (animals, sections being evaluated, number of objects - e.g. airways - being evaluated) must be accurately specified. This should also include information how many animals of a given strain were subjected to further methods, e.g. immunolabeling.

Author’s response: We have amended the results and legends to include the number of animals and slices used, the raw data (i.e. structures with reporter+ fibers out of total structures assessed) for each parameter and (for readability) the percentage of structures with reporter+ fibers. We have also added a new section to the methods detailing the different experimental groups (animals, slices, etc).

2. Characterization of models. The genetic models look elegant and impressive, but as we all know, not each (carefully said) genetic model results in a line that matches our expectation. A thorough characterization of each model is warranted - if that had been given before, an appropriate reference suffices.

Authors’ response: We completely agree that genetic models require extensive characterization and we apologize for not adequately referencing the previous studies with these mice. The TRPV1cre was first characterized in Cavanaugh et al (2011, PMID 21451044), the Tac1cre was first characterized in Harris et al (2014, PMID 25071457), the 5HT3cre was first characterized in Gong et al (2003, PMID 14586460) and the PirtCre was first characterized in Patel et al (2011, PMID 21655234). Importantly, cre-driven reporter expression of these mouse lines in the nodose and jugular vagal ganglia and brainstem have been recently characterized in Kim et al (2020, PMID 32060036) and Su et al (2022, PMID 34755535); indicating that their expression patterns largely match expectation: i.e. pirt-cre is expressed in almost all afferents, TRPV1-cre is expressed in TRPV1-expressing afferents, 5HT3-cre is expressed in nodose-expressing afferents and Tac1-cre is expressed in peptidergic TRPV1+ jugular afferents. These details and references have been added to the text.

3. Controls. The authors used immunolabeling; I have not found a description of methodological controls of this approach.

Authors’ response: We apologize for this omission. We had two different controls which have now been added to the text. First, three of the lung slices of the 5HT3-Ai9 mice were used as controls for immunostaining for E-Cadherin and alpha smooth muscle actin (i.e. the primary antibody was omitted). Secondly, we immunostained for GFP, tdTomato and FLAG in 3 lung slices from 3 TRPV1Cre mice that were not administered AAV9 injections (i.e. they had no reporter expression).

4. Statistics. The authors used student’s t-test throughout (line 266; line 989) without providing evidence of normal distribution of data. Rather, the violin plots depicted in Fig. 2M,N evidently shown a non-normal data distribution, and these data were subjected to t-test. The analyses need to be re-done with non-parametric tests, and precise p-vales rather than “ns” or “*” shall be provided (and, of course, “N"; see above).

Authors’ response: We agree that the data is non-gaussian. We have reanalyzed using non-parametric tests, and added the precise p-values to the text (although some were given by Graphpad as p<0.0001, which is its lower limit). These changes have now identified a significant difference between the diameters of arteries with and without pirt+ innervation. None of the other conclusions have been materially changed.

5. Method details often remain vague and need to be specified. Examples: Line 113: “Specific alleles were confirmed by genotyping per developers’ instructions.” Full details must be made available. Either provide a literature reference, if it was really conducted exactly (!) according to this published protocol, or provide the full protocol here. There are further, less prominent examples, e.g. line 133f “equal volumes”: how much?, and so on. Please work carefully throughput the entire methods description to make sure that description allows others, in principal, to repeat these experiments.

Authors’ response: We have added the methods for the genotyping and have clarified any ambiguities in the text to enable others to repeat our studies.

B. Results

6. It appears as if the authors have overlooked the fact that pulmonary veins in mice (like in other rodents) have an outer sheath of cardiac muscle extending deep into the lung. At these places, a single layer of smooth muscle is located between this outer cardiomyocytes and the endothelium. These veins are innervated, but they are never mentioned in the present manuscript. Fig. 3L,M, 4O and others depict such veins (even the DAPI stain allows to identify the cardiomyocyte nuclei), but the authors speculate about nerve fibers penetrating multiple layers of smooth muscle (rather than the cardiomyocyte sleeve). I recommend to go back to the original material, identify the cardiomyocytes, and then describe innervation of these compartments.

Authors’ response: We thank the reviewer for alerting us to this issue. The comparative physiology review of the structure and composition of pulmonary arteries, capillaries and veins by Mary Townsley (2012, PMID 23606929) describes the biochemical, structural and developmental evidence for cardiomyocytes surrounding extrapulmonary and intrapulmonary airways veins. However, to the best of our knowledge, the most definitive characterization of cardiomyocyte expression on intrapulmonary veins is the work on mouse, rat and human tissue by Mueller-Hoecker et al (2008, PMID 18612369, also referenced by Townsley). This study used h & e staining and a cardiomyocyte-specific mouse line (alphaMHCp-LacZ-hgh) to identify cardiomyocytes. According to the study “a coat of cardiomyocytes was found within pulmonary veins of lungs”. The “cardiomyocytic coat was present in veins measuring 70-250 μm, but not in every vessel of the same size”. Cardiomyocyte coverage was “highly variable not only among specimens, but among veins of the same specimen. Furthermore, in some areas the cardiomyocytic coat was partially incomplete or discontinuous, in particular within lungs”. The authors presented images showing vascular (non-cardiomyocyte) muscle encasing the endothelium with the occasional cardiomyocyte incorporated in this banding (see Fig 2C in Mueller-Hoecker et al). The observed structural variability “may explain the discrepancy of the results of some studies on the existence of a layer of smooth muscle cells between the endothelial lining and the external cardiomyocyte sleeve.” As such, the existence of cardiomyocytes is an incomplete marker of veins (i.e. a cardiomyocyte-free vessel is not necessarily not a vein).

We have gone back over our data sets (including the high magnification versions presented in our original submission) and attempted to identify cardiomyocytes from the DAPI stain with the help of Dr. Sami Noujaim, an expert in cardiomyocyte and pulmonary vein physiology (e.g. PMID 34068960, 30511897, 23559611, 21948246). We unfortunately do not reliably observe the distinct transverse contractile apparatus that should be apparent for cardiomyocytes. Striation-like patterns are visible in the fluorescent images of the muscle, but these are longitudinal and are similar for identified arteries and veins. We believe that only using an appropriate cardiomyocyte-specific immunohistochemical approach would allow for the rigorous detection of this cell type. As such, we are apprehensive to over-interpret our data from the brightfield/DAPI images that we currently have. It is also noteworthy that the ATS official Standard for Quantitative Assessment of Lung Structure (Hsia et al, 2009; PMID 20130146) does not advocate for the use of cardiomyocytes in the detection of veins - rather they advocate using latex injections into the pulmonary vasculature (as we have done for the Pirt-ai9 mice).

Nevertheless, we acknowledge that some of the muscle cells surrounding some of the veins are likely to be cardiomyocytes and thus we have modified the text describing the innervation of the blood vessels and the figures accordingly. Also we are planning future studies to address the hypothesis that cardiomyocytes on intrapulmonary veins represent a key determinant of airway innervation, as they release different growth factors compared to vascular smooth muscle.

7. Neuroendocrine cells of the airway epithelium occur clustered (= neuroepithelial body = NEB) or as solitary cells, and there appear to be differences between NEB and solitary cells. The present authors, however, speak about “NEB” even if only a single cell builds up a “body” (line 498). Please provide information separately for solitary cells and NEB; as the raw data are already collected, this does not require further experimentation

Authors’ response: Of the 117 “NEBs” analyzed only 5 were solitary cells. We have removed these solitary CGRP+ cells and reanalyzed the data. This has not changed our overall conclusions of the NEB data.

C. Writing and intellectual content

8. A line of airway innervation research utilizing elegant genetic models (reporter expression, optogenetics, TRPV1-based cell ablation etc.) is fully neglected here, despite its high relevance to the present study. This also includes a study (PMID: 28002412) addressing the difference between nodose and jugular vagal neurons, very similar to the present study. It is surprising that these prominently published papers - Cell (PMID: 25892222), Nature (PMID: 28002412), Nat Med (PMID: 29505031), PNAS (PMID: 25049382) - are not mentioned here. Instead, the manuscript reads as if this were the first study of airway innervation utilizing such genetic models. These reports need to be adequately addressed in the present manuscript.

Authors’ response: We agree that previous studies have used cre-lox approaches to study the function and neuroanatomy of genetically-defined afferent subsets innervating the airways, and we have now included these studies in the introduction and discussion. We would note that the data presented of the specific lung innervation by afferents labeled by piezo2-cre, Vglut2-cre, P2ry1-cre, Npy2r-cre, Gpr65-cre, wnt1-cre (i.e. neural crest) and phox2b-cre (i.e. placodal) mice in Chang et al and Nonomura et al are individual representative images that do not provide the comprehensive and quantitative analysis that we have attempted here. For the overall mapping, we have instead focused in the present manuscript on the comparison of our data with the excellent study recently published by Su et al (PMID 34755535) which shows in great detail the innervation by specific vagal afferent subsets.

9. Lines 91 to 97 (end of introduction) represent a summary of findings rather than introduction.

Authors’ response: We have omitted this section.

10. The first paragraph of the discussion (lines 519-537) has rather the character of an introduction rather than discussion. In view of the overall length of the discussion, it can be omitted.

Authors’ response: We have moved much of this section to the introduction and in order to incorporate the above references (see reviewer 2, point 8 and reviewer 1, points 3 to 8), we have substantially reorganized the introduction and the discussion.

11. Three times, the authors use “data not shown” for discussing their findings (lines 556, 704, 710). This should be avoided: If these data are relevant, shown them (or don’t draw conclusions out of them).

Authors’ response: We have detailed the number of vagal and DRG neurons labeled by rAAV2 instillation into the lung. The other “not shown” sections have been removed.

Minor

12. The term “alveolar space” is generally used for the alveolar lumen filled with air and lined with surfactant. It is not innervated. Yet, phrases such as “projected out into the alveolar space” (line 357) are commonly used in the text. It shall be replaced by “alveolar region", “alveolar septa” or simply “alveoli", or something similar.

Authors’ response: Corrected.

13. Table 1: Secondary antibodies are not directed against animals (e.g. anti-rabbit), but against immunoglobulins (e.g. anti-rabbit Ig, and so on).

Authors’ response: Corrected.
